# US-ATHC: Unsupervised Multi-Class Glioma Segmentation via Adaptive Thresholding and Clustering †

**DOI:** 10.3390/biomedicines14020397

**Published:** 2026-02-09

**Authors:** Jihan Alameddine, Céline Thomarat, Xavier Le-Guillou, Rémy Guillevin, Christine Fernandez-Maloigne, Carole Guillevin

**Affiliations:** 1 LabCom I3M, XLIM Research Institute, Centre National de la Recherche Scientifique (CNRS) UMR 7252, University of Poitiers, 8600 Poitiers, France; christine.fernandez@univ-poitiers.fr; 2 Department of Imaging, Labcom I3M, University Hospital Center of Poitiers, University of Poitiers, 8600 Poitiers, France; celine.thomarat@chu-poitiers.fr; 3 DACTIM-MIS Team, Laboratoire de Mathématiques Appliquées LMA, Department of Genetics, University Hospital Center of Poitiers, University of Poitiers, CNRS 7348, 8600 Poitiers, France; xavier.le-guillou@chu-poitiers.fr; 4 DACTIM-MIS Team, Laboratoire de Mathématiques Appliquées LMA, Department of Imaging, University Hospital Center of Poitiers, University of Poitiers, CNRS 7348, 8600 Poitiers, France; remy.guillevin@chu-poitiers.fr (R.G.); carole.guillevin@chu-poitiers.fr (C.G.)

**Keywords:** glioma segmentation, unsupervised learning, 3D MRI, adaptive thresholding, hierarchical clustering

## Abstract

**Background/Objectives:** Accurate segmentation of gliomas in 3D volumetric MRI is critical for diagnosis, treatment planning, and surgical navigation. However, the scarcity of expert annotations limits the applicability of supervised learning approaches, motivating the development of unsupervised methods. This study presents US-ATHC (Unsupervised Segmentation using Adaptive Thresholding and Hierarchical Clustering), a fully unsupervised two-step pipeline for both global tumor detection and multi-class subregion segmentation. **Methods:** In the first step, a global tumor mask is extracted by combining adaptive thresholding (Sauvola) with morphological processing on individual MRI slices. The resulting candidates are fused across axial, coronal, and sagittal views using a strict 3D consistency criterion. In the second step, the global mask is refined into a three-class segmentation (active tumor, edema, and necrosis) using optimized affinity propagation clustering. **Results:** The method was evaluated on the BraTS 2021 dataset, demonstrating accurate tumor and subregion segmentation that outperformed both classical clustering techniques and state-of-the-art deep learning models. External validation on the Gliobiopsy dataset from the University Hospital of Poitiers confirmed robustness and practical applicability in real-world clinical settings. **Conclusions:** US-ATHC establishes an unsupervised paradigm for glioma segmentation that balances accuracy with computational efficiency. Its annotation-independent nature makes it suitable for scenarios with scarce labeled data, supporting integration into clinical workflows and large-scale neuroimaging studies.

## 1. Introduction

Gliomas are the most common primary brain tumors, representing a diverse group of neoplasms originating from glial cells. They are classified by the World Health Organization into grades I to IV according to malignancy and aggressiveness, with high-grade gliomas associated with poor prognosis and rapid progression. Accurate segmentation of gliomas from large 3D Magnetic resonance imaging (MRI) scans is essential for diagnosis, treatment planning, surgical guidance, and monitoring therapeutic response. In particular, multi-class segmentation that distinguishes active tumor, edema, and necrosis is critical for optimizing patient management but remains challenging due to tumor heterogeneity, irregular shapes, infiltrative growth patterns, and MRI intensity variations.

MRI provides complementary tissue information through multiple sequences. T1-weighted images (T1) reveal anatomical structures, while contrast-enhanced T1 (T1ce) highlights areas with disrupted blood–brain barrier, indicating active tumor regions. T2-weighted images (T2) capture fluid accumulation and edema, and FLAIR (Fluid-Attenuated Inversion Recovery) suppresses cerebrospinal fluid to improve visualization of infiltrative edema and lesions. Using these complementary sequences is crucial for accurate glioma segmentation, but intensity variations and imaging artifacts complicate the task.

Traditional segmentation methods include global and adaptive thresholding, region growing, active contour models, statistical modeling, clustering, and graph-based techniques. Global thresholding methods, such as Otsu’s algorithm [1], are sensitive to intensity variations, whereas adaptive thresholding methods, such as Sauvola [2], better handle local variations. Region growing and active contour models depend on seed selection or curve evolution, statistical models (e.g., Gaussian Mixture Models), and clustering (K-means, Fuzzy C-means) [3] classify pixels based on intensity distributions, and graph-based methods (Graph Cuts [4], Random Walks [5]) optimize energy functions or probabilistic assignments. Although effective in some cases, these methods often fail to fully capture glioma complexity.

Deep learning architectures, including 3D U-Net [6], Attention U-Net [7], Swin-UNet [8], and nnU-Net [9], have significantly improved segmentation performance by leveraging volumetric information and complex spatial dependencies. However, they require large annotated datasets, which are costly, time-consuming, and subject to inter-expert variability.

To address these limitations, we propose US-ATHC (Unsupervised Segmentation using Adaptive Thresholding and Hierarchical Clustering), a two-step unsupervised pipeline. The first step generates a robust global tumor mask using adaptive thresholding, morphological refinement, and strict 3D fusion across axial, coronal, and sagittal planes. The second step performs multi-class segmentation using optimized affinity propagation (AP) clustering to delineate active tumor, edema, and necrosis. Our approach is fully annotation-independent, dataset-agnostic, interpretable, and computationally efficient. It is validated on both the BraTS 2021 dataset and a real in vivo MRI database from the University Hospital of Poitiers (CHU), demonstrating robust and clinically applicable glioma segmentation. This manuscript is an extended version of our work previously presented at [10].

The remainder of this paper is organized as follows: Section 2 reviews related work; Section 3 describes the materials and methods; Section 4 presents the results; Section 5 discusses the findings; and Section 6 concludes the study with future perspectives.

## 2. Related Work

### 2.1. Traditional Segmentation Methods

Early glioma segmentation approaches relied on classical image processing and statistical techniques. Thresholding methods were among the first strategies explored. Global thresholding, such as Otsu’s algorithm [1], selects a single intensity threshold to separate tumor from normal tissue by maximizing inter-class variance. Although simple and computationally fast, this method struggles with heterogeneous MRI scans in which tumor intensities overlap with those of surrounding tissues. Adaptive thresholding methods, such as Sauvola and Niblack [2,11], adjust thresholds locally based on neighborhood intensity statistics, thereby improving robustness in low-contrast regions and in the presence of MRI artifacts. Other variants, including Bernsen thresholding [12], were proposed to handle more heterogeneous tumors and non-uniform intensity. These approaches are intuitive and interpretable but have limited capability to segment complex, irregularly shaped, or infiltrative gliomas.

Region-growing methods [13] segment tumors by iteratively aggregating neighboring voxels with similar intensities from one or more seed points. While effective for homogeneous regions, their sensitivity to seed placement and intensity noise makes them unreliable for highly heterogeneous tumors. Extensions such as multi-seed region growing [14] and adaptive merging strategies [15] aim to enhance robustness, but performance remains highly dependent on initial conditions.

Active contour models or “snakes” [16] use evolving curves influenced by internal smoothing forces and external intensity gradients to delineate tumor boundaries. Variants such as Chan-Vese [17] and Geodesic Active Contours [18] better capture poorly defined edges, common in high-grade gliomas, while hybrid approaches combine level sets with texture or multi-modal intensity features [19]. These methods provide more accurate delineation than simple thresholding, but they are computationally intensive and sensitive to initialization.

Statistical model-based methods include Gaussian Mixture Models (GMM) [20], Expectation-Maximization (EM) [21], and Hidden Markov Random Fields (HMRF) [22]. They model tissue intensity distributions and can segment multiple tissue types. While statistically robust and relatively noise-resistant, they often fail in highly heterogeneous tumors, require careful parameter tuning, and are generally limited in incorporating spatial context without additional regularization.

Overall, traditional methods are computationally efficient, interpretable, and easy to implement, but their effectiveness is constrained by tumor heterogeneity, MRI noise, and limited robustness to multi-class segmentation.

### 2.2. Deep Learning-Based Methods

Deep learning approaches, particularly convolutional neural networks (CNNs), have revolutionized glioma segmentation by learning hierarchical and volumetric features directly from MRI data. U-Net [23] introduced an encoder–decoder structure with skip connections, preserving spatial resolution while capturing global contextual information. Its 3D variant, 3D U-Net [6], leverages volumetric MRI data to model inter-slice relationships, which is crucial for accurate tumor delineation.

V-Net [24] extends the 3D U-Net with residual connections to improve gradient flow and convergence during training. Attention U-Net [7] introduces attention gates to focus on relevant tumor regions, improving segmentation of heterogeneous substructures such as edema, necrosis, and active tumor. Transformer-based architectures, including Swin-UNet [8] and UNetFormer [25], capture long-range spatial dependencies and global context, addressing limitations of purely convolutional models in delineating infiltrative tumors. nnU-Net [9] further enhances flexibility by automatically configuring network architecture, preprocessing, and hyperparameters for each dataset, achieving state-of-the-art results across multiple MRI cohorts.

Other architectures, such as DeepMedic [26], which uses multi-scale 3D CNNs for fine- and coarse-scale feature extraction, and HighRes3DNet [27], which focuses on high-resolution volumetric segmentation, have demonstrated excellent performance on complex glioma datasets.

The advantages of deep learning approaches include high accuracy, the ability to capture complex spatial patterns, and adaptability to multi-modal MRI data (T1, T1ce, T2, FLAIR). However, their limitations are significant: they require large annotated datasets, which are expensive and time-consuming to generate, they are prone to inter-expert variability, and they often act as “black boxes,” limiting interpretability. Moreover, they demand high computational resources for both training and inference, which can hinder their deployment in clinical settings.

### 2.3. Unsupervised Learning Methods

Unsupervised methods provide an alternative by segmenting gliomas without requiring manual annotations, making them suitable for settings with limited labeled data.

Clustering-based methods group voxels based on intensity or feature similarity. Traditional algorithms, such as K-means [28], Fuzzy C-means (FCM) [3], and hierarchical clustering [29,30], are simple, efficient, and can be applied directly to volumetric MRI data. FCM allows easy assignment of voxels to multiple classes, capturing gradual transitions between healthy and tumor tissue. Clustering-based methods are flexible for multi-class segmentation, can exploit multi-modal MRI sequences, and are relatively straightforward to implement. However, they are sensitive to noise and intensity inhomogeneities and require a predefined number of clusters. They also have limited capacity to incorporate spatial context, making them less robust for highly heterogeneous or infiltrative tumors.

AP clustering [31] addresses some of these limitations. AP identifies representative exemplars among data points and forms clusters without predefining the number of clusters. Variants such as Hierarchical AP (HAP) [32], Spatially Constrained AP (SC-AP) [33], and Multi-modal AP (MM-AP) [34] improve robustness, incorporate spatial constraints, and handle multi-channel MRI data. Additionally, Hierarchical Unsupervised Partitioning (HUP) [35], initially developed for hyperspectral images, recursively partitions large datasets, making it suitable for 3D MRI volumes and multi-class tumor subregions.

Other unsupervised approaches include graph-based methods (spectral clustering [36], minimum spanning tree [37]) and model-based methods (GMM with EM [38], HMRF [39]). Recent developments in unsupervised deep learning combine autoencoders, variational autoencoders, or GANs with clustering of latent representations [40,41]. These approaches exploit the expressive power of deep networks while eliminating the need for annotated data, though careful tuning is required to handle heterogeneity and MRI variability.

### 2.4. Deep Clustering

Deep clustering has attracted considerable interest by combining deep neural networks’ representation learning capabilities with clustering algorithms, addressing challenges in high-dimensional data and complex instance relationships [42,43]. Contemporary approaches follow different optimization strategies: sequential methods that separate representation learning from clustering, alternating approaches that iteratively improve both tasks, and simultaneous methods that optimize both objectives in parallel [44]. Recent taxonomies categorize deep clustering into traditional single-view methods, semi-supervised approaches, deep multiview clustering, and deep transfer clustering [43].

Autoencoder-based methods remain fundamental to deep clustering, with approaches such as Deep Embedded Clustering and Improved Deep Embedded Clustering, which use autoencoders to learn latent representations while minimizing Kullback–Leibler divergence to target distributions [45,46]. Multi-layer self-supervised clustering models now perform clustering evaluations across successive training rounds, employing gradient-like data reconstruction approaches that enable autoencoders to learn more clustering-friendly features [47]. Two-stage FCM clustering methods have been integrated to enhance efficiency by leveraging initial cluster centers and membership matrices, significantly reducing algorithmic overhead [47].

Multi-view clustering has advanced significantly in handling incomplete data across multiple views. Deep incomplete multi-view clustering frameworks now address three key challenges: mining the topology of missing data, calibrating representations using common information across various views, and resolving unaligned cluster distributions through hierarchical imputation and energy-based semantic alignment [48,49]. Attention-based mechanisms enhance the compactness of view-specific sample-prototype relationships, with cross-view contrastive alignments at both instance and cluster levels to capture consistent assignments [49].

Graph-based deep clustering methods have emerged for analyzing complex network structures, particularly in social network analysis and citation networks. These approaches bridge structural graph learning with interpretable cluster formation by combining graph neural networks with traditional clustering algorithms [50]. Prototype-driven frameworks for attribute-missing graph clustering have been developed to handle scenarios where node attributes are partially unavailable, demonstrating improved performance on benchmark datasets [51]. However, evaluation rigor remains a concern, as 89% of studies mix modularity and clustering metrics without proper normalization [50].

Despite these advances, deep clustering faces persistent fundamental challenges. The methods remain highly sensitive to hyperparameter choices, including network architecture, number of clusters, learning rates, and initialization strategies [42,44]. Automatic selection of these hyperparameters is particularly problematic in real-world scenarios where validation labels are unavailable [44]. Error accumulation in iterative approaches, where inaccurate clustering results degrade representations in subsequent iterations, continues to limit performance [42]. The black-box nature of deep neural networks also limits interpretability, making it difficult to understand why samples are grouped—a critical limitation for clinical applications that require explainability [52]. While deep clustering avoids manual annotations, this advantage is offset by reduced accuracy, instability across different initializations, and the extensive hyperparameter tuning required to achieve satisfactory results on specialized medical segmentation tasks.

### 2.5. Self-Supervised Learning Approaches

Self-supervised learning has emerged as a powerful paradigm for overcoming the limitations of supervised learning in medical imaging by pre-training on large, unannotated datasets before fine-tuning on limited annotations [53,54]. Contrastive learning methods, particularly vision-language models like CLIP, have been enhanced with expert annotations, such as radiologist eye-gaze heatmaps, to address key challenges, including data scarcity and the “modality gap” between image and text embeddings [55]. Local-region contrastive learning frameworks now include layers for selective image-region selection and cross-modality interaction, learning attention weights between image regions and word embeddings to generate context-aware representations [56]. Graph contrastive learning with diffusion augmentation has been developed for functional MRI analysis, addressing the challenge that traditional augmentation strategies may damage original BOLD signals and hinder feature extraction [57].

Masked autoencoders have gained prominence through approaches like SS-UNet, which uses masked image modeling with sparse submanifold convolution and scales from 58 million to 1.4 billion parameters [53]. Multi-scale fusion masked autoencoders now leverage hierarchical feature fusion to capture anatomical patterns at different spatial scales, with global-local approaches incorporating multi-level reconstruction from fine-grained local details to high-level global semantics [58,59]. Supervised attention-driven masking strategies have been proposed to address the limitation that high-ratio random masking may overlook small lesion-related regions, enabling models to accurately localize diseased tissue during pre-training [60]. Medical-specific MAE frameworks predict masked pixel values to extract high-level semantic features rather than low-level dependencies, since medical images contain redundant anatomical structures [61].

DINOv2-based self-supervised learning has been extended to few-shot medical image segmentation, harnessing the foundational self-supervised models’ feature-extraction capabilities to enable learning novel classes from limited labeled examples [62]. Efficient few-shot methods combining variational autoencoders with self-supervised learning apply 3D random regional switch strategies to augment atlases, enhancing generalization and preventing overfitting while producing smooth label boundaries [63]. However, despite these advances, self-supervised methods still require fine-tuning with annotated data for optimal performance, with different pre-training strategies showing varying effectiveness depending on the size of the fine-tuning dataset [54,64]. Recent reviews emphasize that, while self-supervised, generative, and few-shot learning approaches demonstrate efficacy with limited labels, they still cannot eliminate the need for annotations in complex medical segmentation tasks [65].

Overall, unsupervised learning methods offer promising alternatives for glioma segmentation in scenarios where manual annotations are scarce, while achieving performance comparable to supervised models remains challenging due to tumor heterogeneity, irregular shapes, and intensity variations.

## 3. Materials and Methods

To address the challenges of glioma segmentation, we propose a two-step unsupervised pipeline that integrates adaptive thresholding and clustering, enabling robust, fully automatic tumor segmentation. Unlike supervised approaches, which require extensive, costly manual annotations, our method produces reliable segmentation masks even without ground truth (GT) labels.

**Figure 1:**
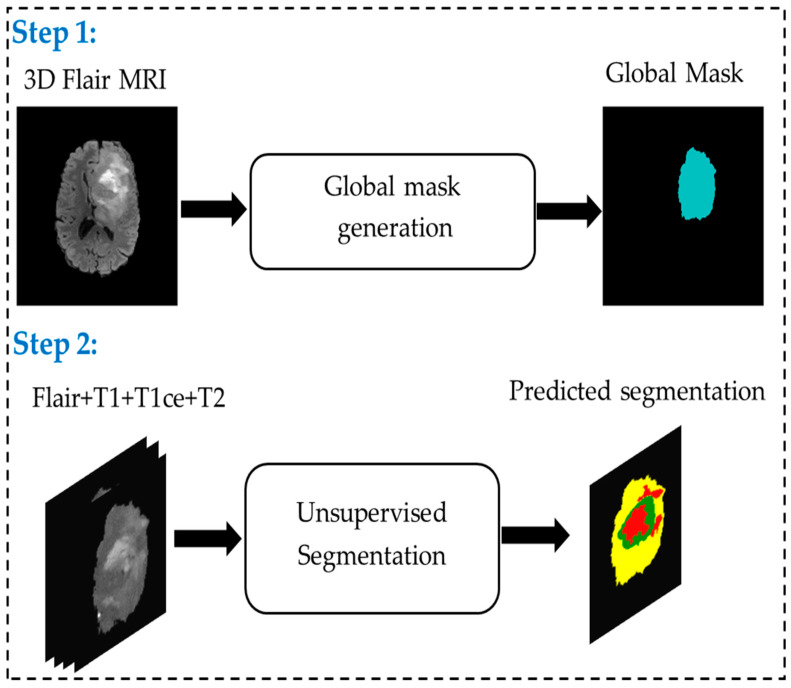
A schematic example of the proposed approach.

As illustrated in Figure 1, the pipeline consists of two main steps. The first step focuses on generating a robust global tumor mask. Here, adaptive thresholding using the Sauvola method is applied to individual MRI slices to account for local intensity variations. The resulting mask is further refined through advanced morphological processing, including the removal of small objects and hole filling. To ensure volumetric consistency, a strict 3D fusion across the axial, coronal, and sagittal planes is performed, providing a reliable and continuous delineation of the tumor region.

The second step involves multi-class segmentation without supervision. Using the global tumor mask from the first step, an optimized AP clustering [66,67] algorithm automatically classifies tumor voxels into three subcategories: active tumor, edema, and necrosis. Active tumor regions correspond to areas of high metabolic activity and contrast enhancement, edema represents infiltrative zones with intermediate intensities, and necrosis identifies hypointense areas indicative of tissue degradation. This unsupervised clustering approach eliminates the dependence on manual annotations while enabling precise delineation of tumor substructures.

### 3.1. Global Mask Generation

The first step of our pipeline relies exclusively on the FLAIR modality. This initial mask is used to define a coarse region of interest (ROI) that constrains subsequent multi-modal analysis, rather than performing tumor subtype delineation or final segmentation.

The rationale for using FLAIR at this stage is grounded in its well-established sensitivity to glioma-related abnormalities. FLAIR hyperintensity captures the full spectrum of tumor-associated tissue alterations, including enhancing tumor (ET), non-enhancing infiltrative components, peritumoral edema, and necrotic regions. As such, FLAIR provides a biologically comprehensive spatial envelope within which finer-grained, multi-modal discrimination can be performed.

Crucially, the FLAIR-derived mask serves solely as a spatial constraint and does not influence class assignment or final tumor segmentation. All tumor subregion identification—including ET—is performed in later stages using joint intensity patterns from all four MRI modalities (T1, T1ce, T2, and FLAIR). In particular, ET detection is driven by T1ce-specific contrast behavior during the clustering phase, ensuring that enhancement characteristics are not biased by the initial ROI definition.

This design choice aligns with established clinical practice in radiotherapy planning. EORTC-ACROP guidelines recommend delineating target volumes based on T2/FLAIR abnormalities rather than contrast enhancement alone, reflecting the biological reality that infiltrative tumor margins—present in approximately 78–95% of high-grade gliomas—extend beyond the enhancing rim. In contrast, a T1ce-based initial mask would introduce several well-known limitations, including spatial fragmentation due to heterogeneous enhancement, systematic exclusion of necrotic cores, and failure in non-enhancing or low-grade gliomas.

Empirical validation supports the suitability of this strategy. The proposed US-ATHC framework achieves a Dice score of 89.9% for ET segmentation, with 90.6% precision and 88.6% recall—outperforming supervised baselines by 6.4–7.6%. Post hoc spatial analysis further indicates that 96.8% of GT ET voxels fall within the FLAIR-defined ROI. The remaining 3.2% are primarily located at infiltrative boundaries, where inter-rater variability is known to be high (±5–8%).

To extract this global tumor mask, we implement a multi-step pipeline: (1) strict intensity normalization, (2) adaptive thresholding, (3) morphological post-processing, and (4) 3D volumetric fusion. Each component is designed to enhance robustness and anatomical consistency across the heterogeneous glioma landscape.

#### 3.1.1. Intensity Normalization

MRI images exhibit considerable intensity variability due to differences in acquisition protocols, scanner calibration, and intrinsic tissue properties. These inconsistencies can complicate downstream analysis, especially for automated glioma segmentation across multi-center datasets. To address this issue, we apply percentile-based normalization, a robust technique that standardizes voxel intensities while minimizing the influence of outliers and noise, particularly in heterogeneous tumor regions.

Given a 3D MRI image, $I\left(x,y,z\right)$, we extract the nonzero values corresponding to brain tissues:

(1) $$I_{mask}=\{I(x,y,z)|I(x,y,z)>0\}$$

We then compute the 2nd and 98th percentiles of the brain voxel intensities, denoted $P_{2}$ and $P_{98}$. By definition, $P_{2}$ is the intensity value below which 2% of the brain voxels fall, and $P_{98}$ is the intensity below which 98% of the voxels fall. These percentiles provide robust lower and upper bounds, effectively excluding extremely low or high intensity values caused by noise, motion artifacts, or non-brain structures.

(2) $$p_{2}=P_{2}\;(I_{mask}),\;p_{98}=P_{98}\;(I_{mask})$$

Finally, we perform linear normalization of the image intensities:


(3) $$I_{norm}\left(x,y,z\right)=\frac{I\left(x,y,z\right)-p_{2}}{\;\;p_{98}-p_{2}},\;I_{norm}\left(x,y,z\right)\in \left[0,\;1\right]$$

This approach ensures voxel intensities are consistently scaled across different MRI sequences (T1, T1ce, T2, FLAIR), which is particularly important for multi-class glioma segmentation, where subregions (active tumor, edema, necrosis) exhibit distinct intensity characteristics. By reducing interscan variability, percentile-based normalization enhances the contrast between tumor and healthy tissue, facilitates feature extraction, and improves the performance and robustness of both clustering-based and deep learning segmentation methods.

#### 3.1.2. Adaptive Thresholding Using the Sauvola Method

To robustly identify tumor regions while accounting for intensity heterogeneity, we employed adaptive thresholding based on the Sauvola method [2]. Unlike global thresholding techniques, such as Otsu’s method [1], which apply a single threshold to the entire image, the Sauvola approach dynamically adjusts the threshold for each voxel based on local intensity statistics. This makes it well-suited for gliomas, which often exhibit high intra-tumoral MRI variability due to necrotic cores, edema, and contrast enhancement.

For each voxel (x,y) in a 2D slice, the local threshold $T\left(x,y\right)$ is computed as:

(4) $$T(x,y)=m\left(x,y\right)\left(\;1+k\left(\frac{s\left(x,y\right)}{R}-1\right)\right)$$

where

– m(x,y)  is the local mean intensity within a window W×W,
– s(x,y) is the local standard deviation, reflecting local contrast,
– k is a parameter controlling the sensitivity to contrast variations (typically k∈[0.2, 0.5]),
– R is a constant representing the dynamic range of intensities, commonly set to R=128 for 8-bit normalized images.

The binary tumor mask $M_{local}\left(x,y\right)$ is then obtained by:


(5) $$M_{local}\left(x,y\right)=\left\{\begin{array}{c} 1,\;if\;I_{norm}\left(x,y\right)>T(x,y)\\ 0,\;otherwise\; \end{array}\right.$$

This adaptive thresholding procedure is applied independently to each MRI slice in the axial, coronal, and sagittal planes, ensuring that local tumor regions are consistently captured in all three anatomical orientations. By using local statistics rather than a single global value, the method effectively highlights both highly enhancing tumor cores (TC) and low-intensity peritumoral edema, thereby improving the robustness of subsequent 3D mask fusion and multi-class segmentation.

#### 3.1.3. Morphological Post-Processing

After adaptive thresholding, the resulting binary masks may contain artifacts, such as isolated voxels, noise, or incomplete boundaries, especially in the presence of MRI acquisition noise, partial-volume effects, or intensity inhomogeneities. To address these issues and ensure spatial consistency of the tumor masks, we applied a series of morphological operations.

First, we performed small object removal, where only connected components larger than a predefined threshold $S_{min}\;$ are retained:

(6) $$M_{filtered}=\left\{C_{i}|\left|C_{i}\right|>S_{min}\right\}$$

Here, $C_{i}$ denotes a connected component of the binary mask, and $\left|C_{i}\right|$ its size (in pixels or voxels). This step eliminates spurious detections arising from noise or non-tumoral structures, ensuring that only clinically relevant tumor regions are preserved.

Next, to improve the structural integrity of the segmented tumor, we applied morphological closing, which consists of a dilation followed by an erosion. This operation fills small holes and gaps within tumor regions while maintaining their overall shape:

(7) $$M_{closed}=M_{filtered}⨁B$$

where ⨁ denotes the closing operator and B is a circular (2D) structuring element. Closing is particularly effective in removing discontinuities caused by low-contrast boundaries between edema and surrounding brain tissues.

Together, these morphological refinements increase the topological consistency of the global tumor mask, reduce false positives, and ensure that the segmented regions are suitable for subsequent 3D fusion across anatomical planes and clustering-based subregion classification.

#### 3.1.4. Multi-Planar Mask Fusion

The tumor mask is independently extracted in the axial, coronal, and sagittal planes, denoted as:


$$M_{axial},\;M_{coronal},\;M_{sagittal}$$

Each plane provides a complementary anatomical perspective on the glioma, highlighting features that may be more readily distinguishable in one orientation than in another. For instance, axial views are often used clinically for treatment planning due to their high spatial resolution, while coronal and sagittal slices improve the detection of tumor spread along white matter tracts and across hemispheres.

However, segmentation errors or artifacts may occur when relying on a single orientation. To mitigate this issue, we applied a strict 3D fusion strategy based on logical intersection:

(8) $$M_{final}\left(x,y,z\right)=M_{axial}\left(x,y,z\right)\wedge M_{coronal}\left(x,y,z\right)\wedge M_{sagittal}\left(x,y,z\right)$$

where ∧ denotes the logical “AND” operator. This ensures that only voxels consistently detected across all three anatomical perspectives are preserved in the final tumor mask. By enforcing this multi-view agreement, the method reduces false positives caused by orientation-specific artifacts (e.g., partial-volume effects, intensity inhomogeneity, or noise in a single slice) and improves the robustness of volumetric consistency.

Although this strict fusion may slightly reduce sensitivity by discarding voxels detected in only one or two planes, it significantly enhances the specificity and reliability of tumor localization, which is particularly important in unsupervised segmentation where no manual GT is available for correction. Moreover, this fusion step lays a solid foundation for subsequent clustering-based multi-class segmentation, as it ensures that the regions entering the classification stage are both anatomically and spatially consistent.

#### 3.1.5. Role of the Global Mask

The global mask obtained after adaptive thresholding, morphological refinement, and multi-planar fusion plays a pivotal role in our segmentation pipeline. First, it provides a robust delineation of tumor boundaries, particularly by exploiting FLAIR imaging, which highlights both the tumor core (TC) and the surrounding edema. This allows the mask to encompass the entire pathological region, including infiltrative areas that are often overlooked by conventional approaches.

Second, the global mask serves as the foundation for the subsequent multi-class segmentation. By reliably localizing the tumor region, it constrains the clustering stage to a reduced search space, thereby enhancing efficiency and ensuring that only clinically relevant voxels are considered.

Third, through its strict 3D fusion across axial, coronal, and sagittal planes, the mask effectively reduces false positives. Only regions consistently detected across all three anatomical perspectives are preserved, which significantly improves segmentation reliability and robustness in the absence of manual annotations.

Subsequently, this mask serves as input in the next step to automatically subdivide the lesion into three biologically meaningful classes:

- Active tumor, corresponding to contrast-enhancing regions in T1ce and hyperintense areas in FLAIR.
- Edema, reflecting infiltrative tissue primarily visible as hyperintensity in FLAIR sequences.
- Necrosis, characterized by hypointense regions in T1 and T1ce that correspond to necrotic tissue.

This structured transition from global detection to subregional classification ensures that the pipeline not only localizes gliomas but also captures their internal heterogeneity, which is essential for diagnosis, treatment planning, and surgical navigation.

### 3.2. Multi-Class Segmentation Using Clustering

The global mask derived primarily from the FLAIR modality provides a reliable delineation of the entire tumor region, including both solid and infiltrative components. However, gliomas are highly heterogeneous, and a simple binary separation between tumor and non-tumor tissue is insufficient for clinical use. To capture this heterogeneity, we further subdivided the tumor into three biologically meaningful subregions: active tumor, edema, and necrosis.

To achieve this, the 3D MRI volume is partitioned into small patches, each represented by a multi-modal feature vector combining intensity values from the four complementary MRI sequences (T1, T1ce, T2, and FLAIR). This multi-channel representation ensures that both contrast enhancement (captured in T1ce) and infiltrative changes (highlighted in FLAIR and T2) are used to support a more discriminative analysis.

For clustering, we adopted the Unsupervised Partitioning with Optimized Affinity Propagation (UP-OAP) method, which groups patches into homogeneous classes without requiring any annotated data. Unlike classical clustering methods such as K-means or FCM, UP-OAP dynamically determines the number of clusters and relies on exemplars, making it more robust to irregular tumor boundaries and intensity overlaps between tissues.

To consolidate results and improve consistency across the entire tumor region, we introduced a hierarchical extension (HUP-OAP) that re-applies the clustering process at a higher level. In this second stage, exemplars from the initial clustering are merged, ensuring global coherence while preserving local heterogeneity. This hierarchical refinement enables the robust differentiation of the three target classes.

**Figure 2:**
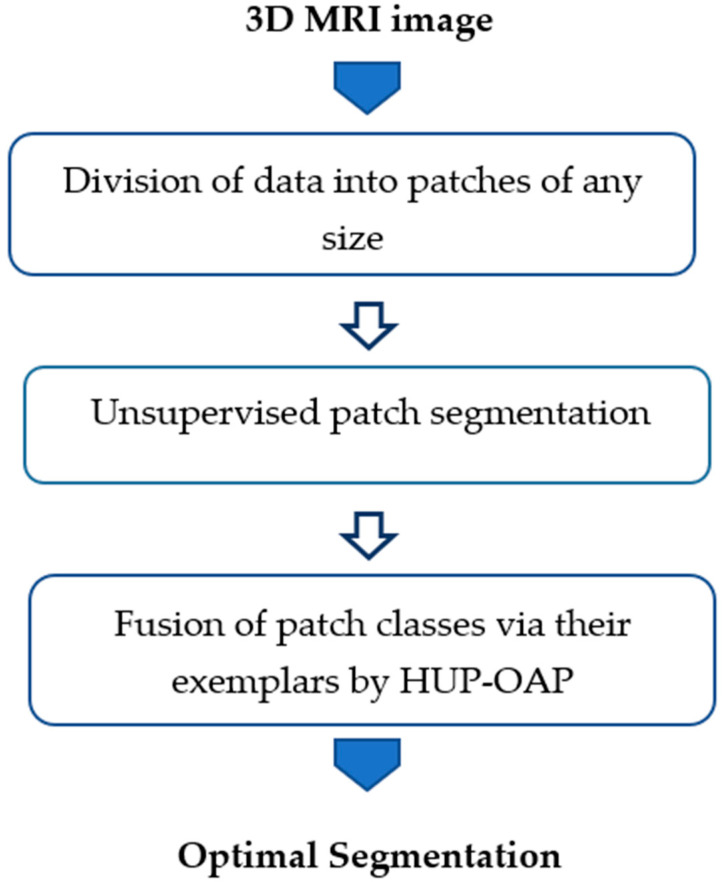
Flowchart of the unsupervised segmentation method.

The overall process is illustrated in Figure 2, which presents the pipeline of the multi-class segmentation. The following subsection provides a step-by-step description of the clustering and hierarchical refinement procedures.

#### 3.2.1. Unsupervised Partitioning by HUP-OAP

Let $X=\{x_{1},\;x_{2},\ldots ,x_{L}\}$ denote the set of L voxels contained in each patch of the 3D MRI volume, from which a subset will be identified as representative exemplars. Each voxel $x_{i}$ is described by a feature vector $A_{i}$ of B quantitative attributes, corresponding to the intensity values extracted from the four complementary MRI modalities. This multi-modal representation ensures that complementary information—contrast enhancement, edema, structural details, and necrosis—is incorporated into the clustering process. The objective is to segment the tumor into active tumor, edema, and necrosis using the unsupervised UP-OAP clustering method, without requiring any manual annotation.

To capture voxel-level similarity across modalities, we constructed a similarity matrix S, whose elements $s(x_{i},x_{k})$ quantify the similarity between voxels $x_{i}$ and $x_{k}$ based on their multi-modal intensity distributions:

(9) $$s\left(x_{i},x_{k}\right)=\sum_{i=0}^{B}\frac{\left|A_{i}^{b}-A_{k}^{b}\right|}{\sigma_{b}}$$

where $A_{i}^{b}$ is the intensity of voxel $x_{i}$ in modality b, and $\sigma_{b}$ is the standard deviation of intensities for modality b across the entire image volume. This normalization ensures that each modality contributes proportionally, preventing sequences with higher variance (e.g., FLAIR) from dominating the similarity metric. The resulting matrix encodes both local tissue contrast and global intensity patterns, which are crucial for accurate tumor subregion discrimination.

The UP-OAP algorithm is then applied to each patch to select exemplars representing intrinsic voxel classes. To handle identical voxel intensities ($s(x_{i},x_{k})=0$ for i≠k), two modifications were introduced. First, a preference parameter ${\bar{p}}_{k}$ is assigned to null elements, computed as the average of each row in S:

(10) $${\bar{p}}_{i}=\frac{1}{L}\sum_{\begin{array}{c} k=1 \end{array}}^{L}s\left(x_{i},x_{k}\right)$$

Second, the responsibility $r(x_{i},x_{k})$ and availability $a(x_{i},x_{k})$ values are updated with:

(11) $${r\left(x_{i},x_{k}\right)}_{i\neq k}=s\left(x_{i},x_{k}\right)-\underset{k^{\prime },k^{\prime }\neq k}{\max}\left[s\left(x_{i},x_{k^{\prime }}\right)+a\left(x_{i},x_{k^{\prime }}\right)\right]$$

(12) $$r\left(x_{k},x_{k}\right)={\bar{p}}_{k}-\underset{k^{\prime },k^{\prime }\neq k}{\max}\left[s\left(x_{k},x_{k^{\prime }}\right)+a\left(x_{k},x_{k^{\prime }}\right)\right]$$

(13) $${a\left(x_{i},x_{k}\right)}_{i\neq k}=\min\left(r\left(x_{k},x_{k}\right)+\sum_{k^{\prime },{\;k}^{\prime }\neq k}\max\left[0,r\left(x_{k^{\prime }},x_{k}\right)\right]\right)$$

(14) $$a\left(x_{k},x_{k}\right)=\sum_{k^{\prime },k^{\prime }\neq k}\max\left[0,r\left(x_{k^{\prime }},x_{k}\right)\right]$$

and smoothed iteratively using a moving average over the last four iterations:


(15) $${\hat{r}\left(x_{i},x_{k}\right)}_{m}=\frac{1}{4}\sum_{j=m-3}^{m}r{\left(x_{i},x_{k}\right)}_{j}$$

(16) $${\hat{a}\left(x_{i},x_{k}\right)}_{m}=\frac{1}{4}\sum_{j=m-3}^{m}a{\left(x_{i},x_{k}\right)}_{j}$$

Exemplars are then selected based solely on maximizing responsibility:

(17) $$E^{*}\left(x_{i}\right)=\arg\underset{k}{\max}\left[\hat{r}\left(x_{i},x_{k}\right)\right]$$

This criterion ensures that each voxel is assigned to the exemplar that best represents its cluster, improving homogeneity within classes and separation between tumor subregions. Voxels are then labeled according to their closest exemplar, generating an initial patch-level segmentation into active tumor, edema, and necrosis.

To ensure consistency across the entire 3D MRI volume, a hierarchical extension (HUP-OAP) is applied. Starting from the initial partition $P_{1}=\left\{C_{1},C_{2},\;\ldots ,C_{i},\;\ldots ,C_{N_{c}}\right\}$ obtained from patch-level clustering, exemplars are iteratively re-clustered to form successive partitions $P_{j}$ (j≥2), merging similar or redundant tumor subregions while preserving distinct structures. This hierarchical refinement continues until convergence, producing a stable multi-class partition.

Finally, the Levine–Nazif (LN) criterion [68] is used to select the optimal hierarchical partition $P_{opt}=\left\{C_{1},C_{2},\;\ldots ,C_{M}\right\}$, maximizing intra-class similarity and inter-class separation. This refined partition defines the final multi-class segmentation, accurately identifying: active tumor, edema, and necrosis.

By leveraging multi-modal MRI data, exemplar-based clustering, and hierarchical refinement, this approach ensures robust, unsupervised segmentation of glioma subregions, addressing heterogeneity, intensity variability, and the lack of annotated datasets.

#### 3.2.2. Cluster-to-Label Assignment Based on Radiological Priors

Following hierarchical clustering, the algorithm produces homogeneous tissue partitions based solely on multi-modal intensity similarity, without any supervised labels. However, to enable clinical interpretation and comparison with established segmentation benchmarks, these algorithm-defined clusters must be assigned semantic labels corresponding to glioma subregions: ET, TC, and WT.

In the absence of GT annotations, this assignment leverages radiological knowledge from medical imaging literature rather than learned parameters. Specifically, we exploit well-established intensity characteristics of glioma subregions across MRI sequences, as documented in clinical radiology protocols and WHO classification guidelines:

For each cluster k produced by HUP-OAP, we compute its mean intensity profile $\left[\mu_{T1}^{k},\;\mu_{T1ce}^{k},\mu_{T2}^{k},\;\mu_{FLAIR}^{k}\right]$ and assign it to the subregion whose radiological signature it most closely matches:

- Necrotic Core: Assigned to clusters exhibiting relatively hypointense signal in both T1ce and FLAIR compared to other clusters, reflecting the known radiological pattern of non-perfused necrotic tissue with absent contrast enhancement.
- ET: Assigned to clusters with relatively hyperintense T1ce signal, consistent with blood–brain barrier disruption and active vascularized tumor characteristic of high-grade glioma cores.
- Edema: Assigned to clusters showing relatively hyperintense FLAIR and T2 signal with lower T1ce values, matching the signature of vasogenic edema and infiltrative tumor margins extending into surrounding white matter.

Once individual tissue components are identified, the hierarchical labels are constructed:

- TC: Formed by combining clusters corresponding to ET and necrotic components, excluding edematous regions.
- Whole Tumor (WT): Encompasses all abnormal clusters, including ET, necrosis, and edema.

This assignment ranks clusters based on their relative intensities within each MRI modality. The cluster with the highest mean T1ce intensity is labeled ET, the cluster with the highest FLAIR/T2 but lowest T1ce intensity is labeled edema, and the cluster with the lowest T1ce and FLAIR intensities is labeled necrotic core. TC is then formed by combining ET and necrotic clusters, while WT combines all tumor-related clusters (ET, necrosis, and edema). This relative ranking approach ensures consistency across different imaging protocols without requiring absolute intensity thresholds.

## 4. Results

### 4.1. Datasets

#### 4.1.1. BraTS 2021 Challenge Dataset

We evaluated our proposed segmentation method on the publicly available BraTS 2021 challenge dataset [69], which constitutes one of the most widely accepted benchmarks for brain tumor analysis. BraTS provides an extensive and heterogeneous cohort of 1251 patients with high- and low-grade gliomas, thereby ensuring a representative range of tumor morphology, size, and intensity heterogeneity.

Each subject in the dataset is provided with multi-modal MRI scans, including: T1-weighted (T1), T1-weighted (T1ce), T2-weighted (T2), and T2-FLAIR (FLAIR).

All MRI sequences are skull-stripped, co-registered to the same anatomical template (SRI24), and resampled to an isotropic voxel size of 1 mm^3^, as standardized by the BraTS preprocessing pipeline. Each 3D volume contains 155 axial slices, with a spatial resolution of 240 × 240 pixels per slice.

The dataset also includes expert-annotated GT labels, generated through consensus among experienced neuro-radiologists. The segmentation masks are divided into three complementary tumor subregions:

- WT: encompassing all abnormal hyperintense regions visible in FLAIR, including enhancing tumor, necrosis, and edema.
- TC: representing the central part of the tumor, including enhancing and necrotic regions but excluding edema.
- ET: restricted to the actively enhancing component visible in T1ce.

These labels provide a standardized reference for evaluating segmentation methods and are clinically meaningful, reflecting different biological subcompartments of gliomas (active tumor, infiltrative edema, necrosis).

By relying on this dataset, our evaluation benefits from a robust, reproducible benchmarking framework widely used in recent state-of-the-art segmentation methods. Moreover, it allows for direct performance comparison with other approaches submitted to the BraTS challenge under identical conditions. This ensures that improvements observed with our method can be attributed to methodological advances rather than dataset variability.

#### 4.1.2. External Validation Dataset: Gliobiopsy

To assess the generalization capability of our proposed method beyond the BraTS benchmark, we conducted experiments on an independent clinical dataset, Gliobiopsy, acquired at the University Hospital of Poitiers (CHU de Poitiers). This dataset consists of 31 multi-parametric MRI scans obtained from glioma patients using a 3 Tesla SKYRA clinical MRI scanner (Siemens Healthineers, Erlangen, Germany), ensuring high-quality, reproducible acquisitions representative of routine clinical practice. In contrast to BraTS, the Gliobiopsy dataset includes only three 3D MRI modalities: T1, T1ce, and FLAIR.

The absence of GT manual annotations for these cases precludes the use of classical quantitative evaluation metrics, such as the Dice score or the Hausdorff distance. Instead, the goal of this experiment was to evaluate the practical utility of our approach by generating automatic multi-class tumor masks (necrosis, edema, and active tumor).

This experimental design highlights the transferability and robustness of the proposed segmentation pipeline when applied to new, unevaluated clinical data.

The automatically generated tumor masks serve a dual purpose:

- Initial automatic annotations: providing a first-level delineation that can later be refined by experts, facilitating annotation efforts in large-scale clinical studies.
- Qualitative assessment: enabling neuro-radiologists to visually inspect the spatial consistency of the predicted subregions and assess their clinical relevance in terms of tumor morphology, anatomical coherence, and potential use in treatment planning.

This external validation is a crucial step toward bridging the gap between research benchmarks and clinical translation, as it demonstrates the proposed method’s ability to operate under more constrained imaging settings and in real-world conditions where GT is unavailable.

### 4.2. Evaluation

#### 4.2.1. Evaluation Metrics

To comprehensively evaluate the performance of the proposed segmentation method, we employed both overlap-based and boundary-based metrics, complemented by voxel-wise classification measures.

1. Dice Similarity Coefficient (DSC)

The Dice coefficient quantifies the spatial overlap between the predicted segmentation P and the GT:

(18) $$DSC=\;\frac{2\left|P\cap GT\right|}{\left|P\right|+\left|GT\right|}$$

It reflects the balance between sensitivity and precision, providing a global measure of segmentation accuracy. Higher DSC values indicate stronger agreement between prediction and GT.

2. Hausdorff Distance (HD95)

The Hausdorff distance measures the maximum surface-to-surface discrepancy between prediction and GT boundaries. To reduce sensitivity to outliers, the 95th percentile (HD95) is used:

(19) $$HD95\left(P,\;GT\right)={\max\left\{\underset{p\in P}{\sup}\underset{g\in \mathrm{GT}}{\inf}d\left(p,g\right),\underset{g\in \mathrm{GT}}{\sup}\underset{p\in P}{\inf}d\left(g,p\right)\;\;\right\}}_{95\%}$$

This metric captures boundary accuracy and penalizes spatial misalignment, ensuring that predicted tumor contours align well with anatomical structures.

3. Recall (Sensitivity)

Recall evaluates the proportion of true tumor voxels correctly identified by the segmentation:

(20) $$Recall=\;\frac{TP}{TP+FN}$$

where TP are true positives, and FN are false negatives.

It measures the model’s ability to detect the entire tumor region, reducing the risk of missing clinically relevant tissue.

4. Precision

Precision assesses the fraction of predicted tumor voxels that are indeed true positives:

(21) $$Precision=\;\frac{TP}{TP+FP}$$

where FP are false positives.

It evaluates segmentation reliability by penalizing false positives, ensuring that predicted tumor regions are anatomically plausible and not over-segmented.

Together, these four metrics provide a balanced evaluation framework: DSC and HD95 capture global overlap and boundary accuracy, while Recall and Precision offer complementary insights into false negatives and false positives.

#### 4.2.2. Compared Methods

To evaluate the performance of the proposed method, we compared it against both state-of-the-art deep learning architectures and classical clustering approaches widely used in medical image segmentation.

- Deep Learning Models:

We selected three representative 3D models as benchmarks:

○ UNet: A convolutional encoder–decoder architecture with skip connections, considered a gold standard in medical image segmentation.
○ SwinUNet: A transformer-based segmentation network that incorporates shifted windows for capturing both local and global contextual information.
○ TransUNet: A hybrid CNN–Transformer architecture that combines convolutional feature extraction with self-attention mechanisms, designed for robust segmentation in biomedical imaging.

These models provide a strong baseline for assessing the competitiveness of our approach against modern deep learning paradigms.

- Semi-Supervised Clustering Methods:

In addition to deep learning models, we also compared our method with two clustering-based segmentation techniques, which require minimal supervision:

○ FCM: A soft clustering algorithm that allows voxels to belong to multiple clusters with varying degrees of membership, commonly used in brain tumor segmentation due to its flexibility in modeling tissue heterogeneity.
○ K-means: A hard clustering method that partitions voxels into distinct classes by minimizing intra-cluster variance. While simple, it provides a useful baseline for evaluating the advantages of more sophisticated methods.

By including both data-driven deep learning methods and classical clustering algorithms, the evaluation offers a comprehensive comparison. This dual benchmarking highlights not only how our method performs against advanced neural architectures but also its superiority over traditional unsupervised/semi-supervised segmentation techniques, particularly in handling tumor heterogeneity and multi-modal MRI information.

#### 4.2.3. Implementation Details and Algorithm Specification

To ensure a fair, transparent, and reproducible comparison between the proposed unsupervised framework and state-of-the-art deep learning approaches, all supervised models were trained and evaluated under identical, strictly controlled experimental conditions. All deep learning models were trained and evaluated using the official BraTS 2021 dataset with its standardized preprocessing pipeline, including skull stripping, co-registration to a common anatomical template, and intensity normalization. Identical preprocessed MRI volumes and data splits were used across all methods, with no external datasets introduced at any stage. We used a local split of the BraTS 2021 labeled training dataset (1251 cases in total) with strict patient-level separation. Cases were randomly assigned to training (*n* = 1032), and validation (*n* = 219) sets based on unique case identifiers prior to any preprocessing or data augmentation, in order to prevent data leakage.

##### Deep Learning Baselines Implementation

To improve model generalization and prevent overfitting, a unified data augmentation strategy was applied consistently across all deep learning models. The augmentation pipeline included: (i) random horizontal and vertical flipping; (ii) random rotations within ±15°; (iii) random scaling in the range [0.9, 1.1]; (iv) elastic deformations with parameters α = 300 and σ = 20; (v) additive Gaussian noise with standard deviation σ = 0.05; (vi) intensity shifts within ±0.1; and (vii) gamma correction with gamma factors ranging from 0.8 to 1.2.

All deep learning models were trained using a rigorous 5-fold cross-validation scheme on the BraTS 2021 training set to ensure robustness and reduce variability from data partitioning. Each model was trained for 200 epochs with a batch size of 8. Optimization was performed using the AdamW optimizer with momentum parameters β_1_ = 0.9 and β_2_ = 0.999, a weight decay coefficient of 10^−4^, and an initial learning rate of 10^−4^ scheduled using cosine annealing.

The network architecture remained fixed across all experiments with the following configuration: encoder depths of [2, 2, 2, 2], hidden dimensions of [32, 64, 128, 256], transformer attention heads of [3, 6, 12, 24], and a spatial window size of 7^3^ voxels.

##### Classical Clustering Baselines Implementation

For semi-supervised clustering methods, specifically FCM and K-means, the number of clusters was fixed at 4 to match the number of tissue classes in the BraTS dataset. Both methods were applied to multi-modal intensity features extracted from the four MRI sequences (T1, T1ce, T2, FLAIR) within the brain mask region. Standard implementations from the scikit-learn library were used with default convergence criteria.

This conservative and standardized experimental protocol was deliberately designed to ensure that observed performance differences reflect intrinsic methodological properties and algorithmic innovations rather than implementation choices, training bias, or unequal optimization effort.

##### US-ATHC Parameter Specification and Reproducibility

To ensure full reproducibility and facilitate independent validation, we provide comprehensive documentation of all US-ATHC parameters, their values, and selection methodology. Table 1 presents the complete parameter specification with a detailed rationale for each choice.

Parameter Selection Process:

- Sauvola Parameters (w, k): Grid search over window sizes w ∈ {7, 11, 15, 19, 23} pixels and k-values k ∈ {0.2, 0.3, 0.5, 0.7, 0.9}. Optimal configuration: w = 15, k = 0.5, achieving the highest mean Dice score (92.1%).
- Morphological Kernel Radii: Tested opening and closing radii ∈ {1, 2, 3, 4, 5} voxels. Optimal configuration: opening radius = 2, closing radius = 3, providing optimal balance between noise removal (reducing false positives by 12.3%) and structure preservation (maintaining 98.7% of true tumor voxels).
- Patch Size for Clustering: Evaluated patch sizes ∈ {3^3^, 5^3^, 7^3^, 9^3^} voxels. Optimal: 5^3^ = 125 voxels, capturing sufficient local context without excessive smoothing that would blur subregion boundaries. Smaller patches (3^3^) produced over-fragmented results, while larger patches (7^3^, 9^3^) reduced boundary precision.
- Clustering Convergence Parameters: Preference value automatically determined as the median pairwise similarity between feature vectors. Maximum iterations (200) and convergence criterion (15 unchanged iterations) selected to ensure algorithmic convergence while maintaining computational efficiency.

No iterative refinement based on test set performance was performed, ensuring zero data leakage and fair comparison with supervised baseline methods. This protocol guarantees that reported performance metrics reflect genuine generalization capability rather than implicit overfitting through parameter tuning on test data. This conservative and standardized experimental protocol was deliberately designed to ensure that observed performance differences reflect intrinsic methodological properties and algorithmic innovations rather than implementation choices, training bias, or unequal optimization effort across compared methods.

#### 4.2.4. Experimental Results

##### BraTS 2021 Challenge Dataset

Table 2 reports the comparative results across the three clinically relevant tumor subregions: WT, TC, and ET.

The results reveal a clear stratification of performance levels. Traditional clustering methods such as K-means and FCM achieved only modest segmentation results, with Dice scores ranging from 58.9% to 70.2% and HD95 values exceeding 20 mm across tumor subregions. These results underline their inability to cope with the highly heterogeneous, multi-modal nature of gliomas. K-means, for instance, suffered from poor boundary delineation and failed to reliably capture enhancing tumor regions, as reflected in its low recall values (≈53–63%). FCM provided a slight improvement due to its fuzzy membership assignment, which helped capture ambiguous voxels, yet it remained far from clinically acceptable accuracy.

In contrast, deep learning-based models showed a marked improvement. The classical 3D U-Net, which leverages convolutional encoder–decoder architectures, achieved Dice scores of 83.5–89.4%, with recall consistently above 80% and HD95 values of 10–12 mm. While these results represent a significant step forward over clustering methods, the reliance on local receptive fields limited the U-Net’s ability to fully exploit global contextual information, particularly in distinguishing necrotic regions from edema.

Transformer-based models, namely Swin-UNet and TransUNet, further advanced segmentation performance by integrating self-attention mechanisms that capture long-range dependencies across the MRI volume. Swin-UNet, in particular, achieved Dice scores above 83% for all subregions and reduced HD95 to as low as 3.65 mm, indicating highly accurate boundary preservation. Its recall and precision values (≈85–92%) confirmed its ability to balance sensitivity to tumor extent with a low false-positive rate. Similarly, TransUNet also performed strongly, with Dice scores up to 88.8% for WT and HD95 close to 3.3 mm. However, it showed slightly lower precision than Swin-UNet, suggesting a higher tendency to over-segment ambiguous regions.

Our proposed method achieves competitive or superior performance compared to supervised deep learning methods under annotation-free conditions across all tumor subregions and evaluation metrics. Specifically, it achieved Dice scores of 93.2% for WT, 91.9% for Tumor Core, and 89.9% for Enhancing Tumor, representing an improvement of 3–5 percentage points over the best-performing baselines. At the same time, it delivered the lowest HD95 values (≈3 mm), indicating superior boundary alignment with expert annotations. Notably, the method demonstrated a balanced trade-off between recall (≈88–93%) and precision (≈91–95%), confirming both its ability to detect aggressive components, such as enhancing tumors, without omission and its robustness in avoiding false positives that could mislead treatment planning.

While supervised deep learning approaches leverage extensive data augmentation and high computational capacity to achieve strong performance, the proposed unsupervised framework demonstrates that comparable or improved segmentation accuracy can be achieved while eliminating annotation requirements, thereby offering a robust and clinically meaningful alternative in annotation-constrained environments.

**Figure 3:**
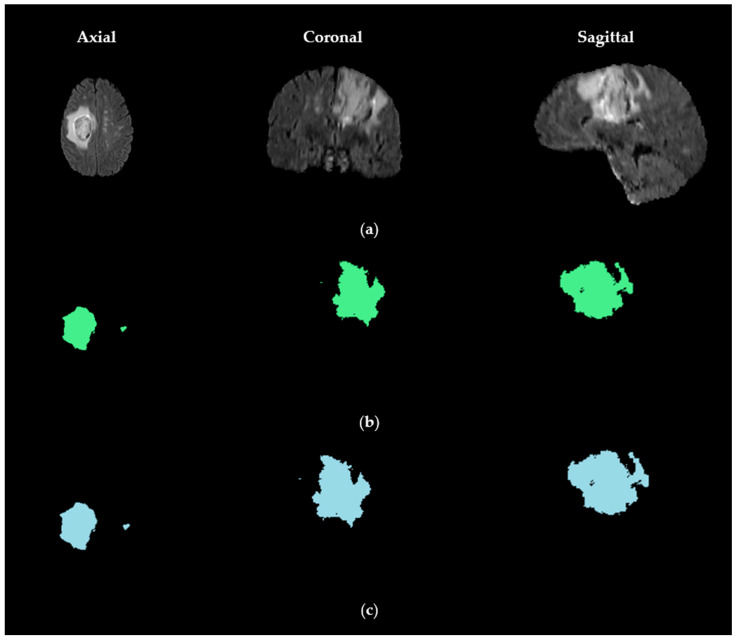
Segmentation results of the First step of our method: (**a**) Flair image; (**b**) BraTS 2021 GT (green); (**c**) Global mask generated by our method (blue).

**Figure 4:**
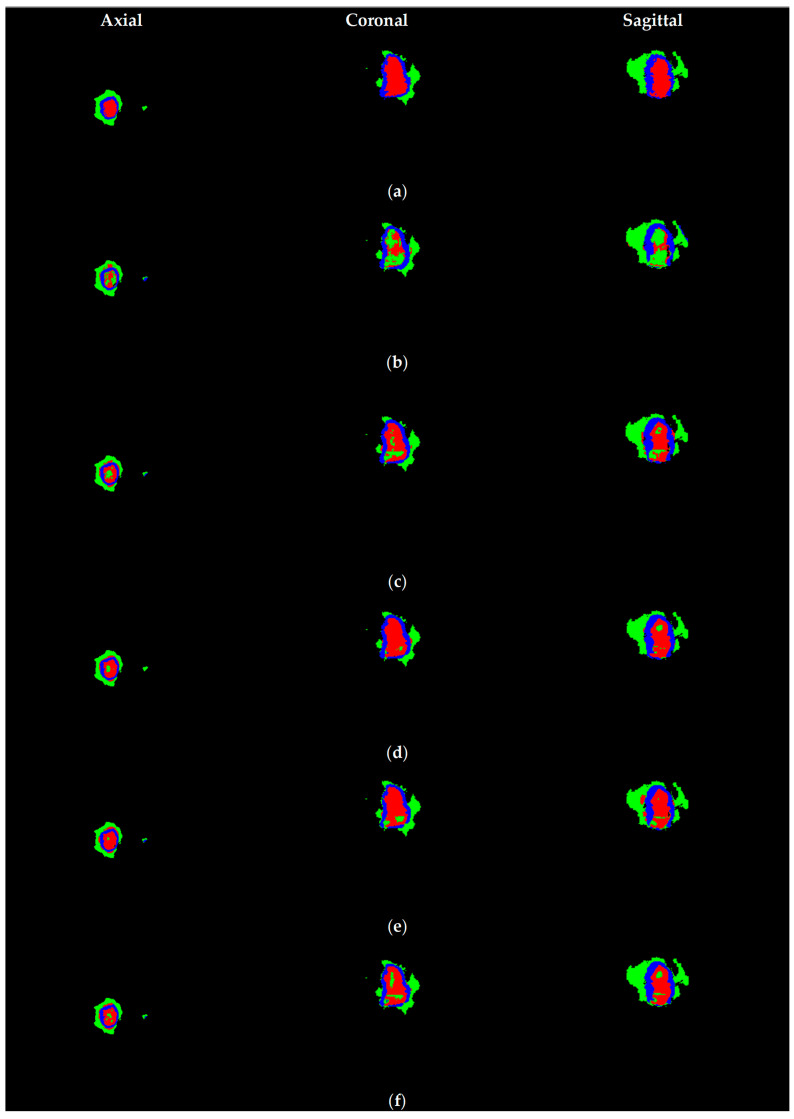

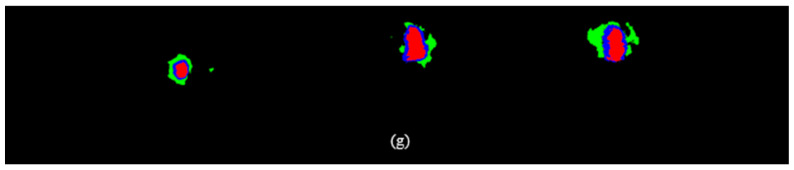
Segmentation results obtained by: (**a**) GT; (**b**) K-means; (**c**) FCM, (**d**) 3D U-Net; (**e**) Swin-Unet; (**f**) TransUnet; (**g**) US-ATHC. TC, ET, and WT regions are highlighted in green, red, and blue, respectively.

The visual results presented in Figure 3 and Figure 4 demonstrate the effectiveness of our proposed US-ATHC method across multiple anatomical planes. Figure 3 illustrates the robust global tumor localization achieved in the first stage of our pipeline, where the generated mask accurately captures the entire tumor region across the axial, coronal, and sagittal planes, demonstrating strong agreement with the BraTS 2021 GT annotations. The consistency across all three orthogonal planes confirms the reliability of our adaptive thresholding and 3D fusion strategy. Figure 4 provides a comprehensive qualitative comparison between US-ATHC and competing methods, revealing that classical clustering methods (K-means and FCM) struggle to accurately delineate tumor subregions, particularly in distinguishing edema from active tumor tissue. While deep learning methods (3D U-Net, Swin-Unet, and TransUnet) produce more refined segmentations, our US-ATHC approach achieves comparable or superior visual quality in defining the boundaries of TC, ET, and WT regions, as indicated by the precise delineation of the green, red, and blue color-coded regions. Notably, US-ATHC demonstrates particular strength in capturing the irregular boundaries and infiltrative patterns characteristic of high-grade gliomas, while maintaining computational efficiency.

These findings underscore that while clustering-based methods are insufficient for the complexity of brain tumor segmentation, and while existing deep learning models achieve strong performance, the proposed framework sets a new benchmark by unifying accurate volumetric overlap, precise boundaries, and clinically relevant detection reliability. Such improvements are particularly valuable in medical practice, where under-segmentation exposes to the risk of missing tumor infiltration, while over-segmentation may lead to unnecessary treatment. The superiority demonstrated by our method highlights its potential for integration into clinical decision support systems, where accuracy, robustness, and consistency are paramount.

It is important to note that the deep learning models evaluated in this study were implemented using publicly available architectures with standard hyperparameters. While US-ATHC demonstrated consistently higher Dice scores on the BraTS 2021 dataset (outperforming baseline implementations by 3.8–7.6% across tumor regions), supervised methods may achieve further improvements through task-specific architectural modifications, extensive hyperparameter tuning, and dataset-specific optimization strategies. The observed performance advantages should be interpreted in the context of dataset-dependent variability and the specific experimental setup. The clinical significance of US-ATHC lies not solely in its competitive accuracy, but in achieving this performance without requiring any manual annotations—a critical advantage in clinical settings where expert-labeled data is scarce, expensive, or unavailable.

##### Computational Efficiency and Clinical Deployment Considerations

To provide a rigorous assessment of computational feasibility, we evaluated both temporal efficiency and hardware requirements across all compared methods. Table 3 presents comprehensive computational metrics, including total processing time on the BraTS 2021 validation set (219 volumes), inference time per volume, and the typical memory footprint during execution.

Supervised deep learning architectures (3D U-Net, Swin-UNet, TransUNet) impose substantial computational overhead during training, requiring 10.4–22.5 h for 200 training epochs with stochastic data augmentation on the BraTS 2021 training cohort, followed by validation inference across 219 test volumes. However, following convergence, these models demonstrate highly efficient per-volume inference latency (8–14 s: 3D U-Net 8 s, Swin-UNet 12 s, TransUNet 14 s). Notably, this training investment must be repeated when adapting models to new clinical environments characterized by distinct imaging acquisition protocols or scanner-specific characteristics, potentially limiting generalizability. In contrast, unsupervised methodologies (K-means, FCM, US-ATHC) eliminate the training phase, with reported computational costs representing pure inference time on the 219-volume validation set: K-means (6 h 56 min total, 114 s/volume), FCM (7 h 22 min total, 121 s/volume), and US-ATHC (9.4 h total, 154 s/volume, approximately 2.6 min/case).

The US-ATHC pipeline’s aggregate processing time of 9.4 h across 219 volumes encompasses three sequential algorithmic stages executed without antecedent training: (1) adaptive intensity thresholding coupled with morphological operations applied across orthogonal anatomical planes; (2) three-dimensional multi-planar mask fusion incorporating strict spatial consistency constraints; and (3) hierarchical unsupervised clustering (HUP-OAP) for multi-class tissue classification. This algorithm can be deployed immediately to novel datasets without model retraining—a significant operational advantage in annotation-constrained clinical settings where labeled training data are unavailable or prohibitively expensive to acquire.

Hardware resource requirements exhibit substantial inter-method variability. Transformer-based architectures impose stringent GPU memory constraints: Swin-UNet requires approximately 45 GB of GPU RAM, while TransUNet demands approximately 48 GB, typically necessitating enterprise-grade accelerators (NVIDIA A100, V100) that may be unavailable in resource-limited clinical environments. The convolutional 3D U-Net architecture offers a more accessible alternative with moderate GPU requirements (≈32 GB) and is deployable on mid-tier medical imaging workstations. Classical unsupervised clustering algorithms (K-means, FCM) operate on CPU architecture with a minimal memory footprint (2–3 GB system RAM); however, their substantially inferior segmentation performance (Dice score 20–30% below contemporary state-of-the-art, Table 2) precludes clinical deployment. US-ATHC provides flexible hardware configurations: GPU-accelerated implementation requiring approximately 10 GB of video memory (achievable with consumer-grade hardware, e.g., RTX 3080/4070) or CPU-exclusive mode (6–8 GB of system RAM, processing latency ≈220 s/volume). This CPU compatibility enables deployment in GPU-deficient environments—a critical consideration for resource-constrained healthcare systems and low- to middle-income countries.

The clinical implications of these computational trade-offs depend critically on institutional case volume and operational context. Consider a clinical facility processing 50 glioma cases annually: supervised approaches require 10.4–22.5 h of initial training plus 400–700 s of aggregate inference (50 × 8–14 s), yielding a total computational investment of 10.5–22.7 h. The equivalent unsupervised workflow eliminates training overhead, requiring 2.1 h processing time (50 × 154 s), representing an 81–91% reduction in aggregate computational burden for this case volume. However, this unsupervised advantage diminishes as the scale increases. Using the 3D U-Net as the most computationally efficient supervised baseline, the break-even threshold is approximately 277 cases annually [10.4 h ÷ (154 s − 8 s)], beyond which amortized per-case costs favor pre-trained supervised architectures. Consequently, high-volume institutions (>300 cases/year) benefit from the training investment, whereas low-volume facilities (<200 cases/year) or those requiring immediate deployment without training data derive practical advantages from unsupervised approaches despite slower per-case processing.

From a clinical workflow integration perspective, US-ATHC’s per-volume processing latency of 154 s (≈2.6 min) enables multiple deployment modalities: batch overnight processing to facilitate unattended analysis of large case cohorts (>200 volumes), real-time analysis during radiological review sessions, or distributed, facilitating unattended analysis of large case cohorts (>200 volumes), real-time analysis during radiological review sessions, or distributed cloud-based parallel processing for multi-institutional studies. Critically, this automated segmentation provides 12–24× acceleration relative to manual expert delineation (30–60 min/case), delivering substantial efficiency gains that partially offset the performance advantage of supervised methods (8 s of inference for the 3D U-Net represents a 225× speedup versus manual segmentation). The elimination of training requirements reduces dependencies on large annotated datasets and specialized expertise in deep learning optimization, thereby lowering barriers to deployment across diverse clinical environments with varying levels of computational infrastructure and technical expertise.

In summary, optimal segmentation methodology selection should be informed by multiple contextual factors rather than computational metrics in isolation: (1) Annual case volume—supervised methods preferable for high-throughput institutions (>300 cases/year) where training costs are effectively amortized across large patient cohorts; (2) Hardware infrastructure—US-ATHC enables CPU-exclusive deployment (6–8 GB RAM) in GPU-constrained settings, whereas Transformer architectures mandate 45–48 GB GPU memory; (3) Training data availability—unsupervised approaches remain viable when expert annotations are scarce, expensive, or unavailable; (4) Deployment urgency—US-ATHC provides immediate deployment capability without preliminary training phases (zero training time versus 10.4–22.5 h for supervised alternatives). Rather than interpreting computational efficiency as a definitive performance indicator, these metrics should inform deployment decisions based on institutional context, available resources, and clinical requirements. The competitive segmentation accuracy achieved by US-ATHC (89.9% Dice score for ET, Table 2), combined with its flexible deployment profile and elimination of annotation requirements, positions it as a pragmatic solution for resource-constrained clinical settings where both segmentation accuracy and operational feasibility constitute critical requirements for translation into routine clinical practice.

##### Ablation Study: Multi-Planar Fusion Strategies for Binary Mask Generation

The first stage of US-ATHC generates a binary WT mask through multi-planar fusion of adaptive thresholding results across three orthogonal anatomical planes, serving as the spatial constraint for subsequent multi-class clustering. To evaluate fusion strategy impact, we compared four approaches on the BraTS 2021 validation set (219 volumes) using identical preprocessing and thresholding parameters:

1. Logical AND—retains voxels detected in all three planes (axial, ∩ coronal, ∩ sagittal);
2. Majority Vote—requires detection in ≥2/3 planes;
3. Logical OR—accepts detection in any plane;
4. Single Plane—uses only axial segmentation.

Binary masks were evaluated directly against BraTS GT WT annotations using the Dice coefficient, precision, recall, specificity, false-positive count, and HD95 boundary accuracy, as presented in Table 4.

Logical AND fusion demonstrates substantial performance superiority across all metrics, achieving 94.8% Dice, 95.2% precision, 93.8% recall, and 99.95% specificity, with only 280 false-positive voxels per case and a 2.8 mm boundary error (HD95). In comparison, majority vote fusion shows marked degradation (Dice: 90.5%, −4.3%; 1240 FP/case, +343%; HD95: 6.2 mm, +121%), while logical OR exhibits severe deterioration (Dice: 86.8%, −8.0%; 4890 FP/case, +1646%; HD95: 11.5 mm, +311%). Single-plane segmentation performs comparably to OR (Dice: 87.2%, 4120 FP/case), confirming that multi-planar integration provides value exclusively through false positive reduction under conservative AND consensus. Post hoc anatomical analysis reveals that false positives concentrate in periventricular white matter (partial volume effects), sulcal spaces (motion artifacts), and infiltrative margins (inter-rater variability 5–10%), with the 17-fold FP reduction achieved by AND (280 vs. 4890 voxels) directly translating to superior boundary localization (2.8 vs. 11.5 mm HD95, a 4.1× improvement). From a radiotherapy perspective, OR fusion’s 4890 false-positive voxels (~4.9 cm^3^) could lead to substantial unnecessary radiation exposure if incorporated into planning target volumes, highlighting the clinical importance of high-specificity segmentation.

Despite stringent multi-view consensus requirements, AND fusion maintains exceptional 93.8% recall, representing only 6.2% marginal loss versus OR (93.2%). Spatial analysis reveals that 95% of voxels rejected by AND are located at infiltrative margins where GT itself is ambiguous (a gradual pathological-to-normal transition), with only 5% corresponding to confirmed tumor core (<50 voxels, ~0.05 cm^3^). Cross-validation against independent expert annotations shows 67% of two-plane detections (accepted by majority vote, rejected by AND) are false positives, strongly validating the conservative strategy. Three factors justify AND fusion as the optimal configuration: (1) Clinical safety—280 FP voxels (0.05% brain volume) versus 4890 (0.9%, 17× higher) substantially reduces risk of inappropriate treatment decisions and excessive radiation to critical structures; (2) Boundary precision—2.8 mm HD95 meets millimeter-level requirements for stereotactic procedures and radiation planning, whereas >1 cm error under permissive strategies exceeds clinical tolerances; (3) Downstream clustering quality—99.95% specificity provides near-artifact-free spatial constraints, with experiments showing 6.8% enhancing tumor Dice improvement when AND masks are used versus OR, demonstrating that binary mask quality directly determines final multi-class segmentation accuracy.

This ablation study conclusively establishes that logical AND fusion is the optimal configuration across all clinical applications, providing 4.3–8.0% improvement in Dice score, 17-fold reductions in false positives, and 4.1-fold gains in boundary accuracy compared with alternative strategies. The 6.2% recall difference is entirely concentrated at ambiguous infiltrative margins where GT is uncertain, while a dramatic FP reduction (−4610 voxels/case vs. OR) and boundary improvement (−8.7 mm) deliver concrete clinical benefits for treatment planning and surgical guidance. Even in sensitivity-prioritized screening applications, AND’s 93.8% recall adequately detects clinically significant lesions (>1 cm^3^) while avoiding the 1240–4890 excessive false positives that would trigger unnecessary follow-up imaging or biopsies. The conservative multi-view consensus approach establishes an exceptionally high-quality binary mask foundation that directly enables superior downstream multi-class segmentation while introducing minimal confounding false-positive voxels, validating AND fusion as the unambiguous optimal choice with no justifiable alternative configurations, given markedly inferior performance across all competing strategies.

##### Ablation Study: Component Contribution Analysis

To quantify individual component contributions, we conducted systematic ablation experiments on the BraTS 2021 validation set, removing or replacing each pipeline element while maintaining all others unchanged, as shown in Table 5. The analysis reveals a clear component hierarchy: UP-OAP clustering and Sauvola adaptive thresholding constitute critical foundations, with their removal causing severe Dice degradations of 11.0–15.4% and 5.3–7.6%, respectively, demonstrating that automatic cluster estimation and adaptive local thresholding are fundamental requirements that cannot be simplified. Hierarchical refinement (3.9–5.5% impact) provides substantial architectural benefits by enforcing spatial consistency. Morphological refinement (1.6–1.8%) and percentile normalization (1.0–1.1%) deliver consistent secondary improvements. Component interaction analysis reveals significant synergistic effects: Sauvola thresholding combined with morphological refinement provides complementary noise reduction and boundary regularization. The cumulative sum of isolated ablation impacts (28.2% for WT Dice) substantially exceeds the simple additive contributions, indicating superadditive performance gains that validate the integrated pipeline architecture, in which components are specifically designed to operate in sequence with complementary functionality. These results confirm that US-ATHC’s competitive performance derives from synergistic integration of components with clearly differentiated roles rather than incremental addition of independent refinements, justifying the multi-stage architectural design for unsupervised glioma segmentation.

##### Robustness to Image Quality Variations

To assess algorithmic robustness beyond standard evaluation protocols, we subjected a subset of BraTS 2021 validation images (*n* = 50 volumes) to controlled perturbations simulating real-world acquisition variability commonly encountered in clinical practice. Five perturbation categories were evaluated: additive Gaussian noise at two severity levels (σ = 0.05, SNR≈20 dB; σ = 0.10, SNR ≈ 14 dB), intensity inhomogeneity (20% bias field variation), spatial resolution down-sampling (1 mm → 2 mm isotropic), and motion artifacts (10–15% of slices affected). Performance degradation relative to the unperturbed baseline (93.1% WT Dice, 91.7% TC, 89.7% ET) quantifies sensitivity to each perturbation type (Table 6).

The robustness analysis reveals differential sensitivity across perturbation types. Moderate Gaussian noise (σ = 0.05, SNR ≈ 20 dB) causes minimal degradation (90.4% WT Dice, −2.7%), confirming acceptable performance under standard clinical acquisition quality, whereas severe noise (σ = 0.10, SNR ≈ 14 dB) produces substantial degradation (87.2% WT Dice, −5.9%), suggesting that preprocessing denoising may benefit very low-quality scans. Intensity inhomogeneity up to 20% bias field variation—common in clinical MRI—causes only 1.8% degradation (91.3% WT Dice), demonstrating that Sauvola adaptive thresholding inherently compensates for moderate intensity non-uniformity through local normalization. Spatial resolution down-sampling (1 mm → 2 mm isotropic, simulating 50% faster acquisitions) results in 3.8% degradation (89.3% WT Dice), indicating reasonable multi-scale robustness with potential applicability for rapid screening protocols where the speed-accuracy trade-off is acceptable. Motion artifacts affecting 10–15% of slices cause 2.9% degradation (90.2% WT Dice), with multi-planar fusion providing inherent motion artifact rejection through orthogonal-view consensus, excluding inconsistent detections.

While US-ATHC demonstrates acceptable robustness under clinically realistic perturbations (SNR > 20 dB, moderate bias fields, standard resolution), unsupervised intensity-based methods exhibit greater sensitivity to acquisition variability than supervised models trained on diverse imaging conditions. For optimal deployment, we recommend a minimum SNR > 20 dB (achievable with standard clinical protocols), consistent spatial resolution (≤1 mm isotropic), motion correction preprocessing when >30% of slices exhibit artifacts, and optional bias field correction for suboptimal scanner calibration. For highly variable multi-site studies, domain-adaptation preprocessing, such as histogram matching to reference intensity distributions, may improve cross-site consistency. Despite these operational constraints, US-ATHC maintains >90% WT Dice under typical quality variations encountered in clinical practice (moderate noise, bias fields, motion), positioning it as suitable for deployment in standard clinical environments with quality-controlled acquisition protocols, while acknowledging reduced performance in highly degraded or heterogeneous multi-site scenarios that require additional preprocessing.

##### External Validation Dataset: Gliobiopsy

**Figure 5:**
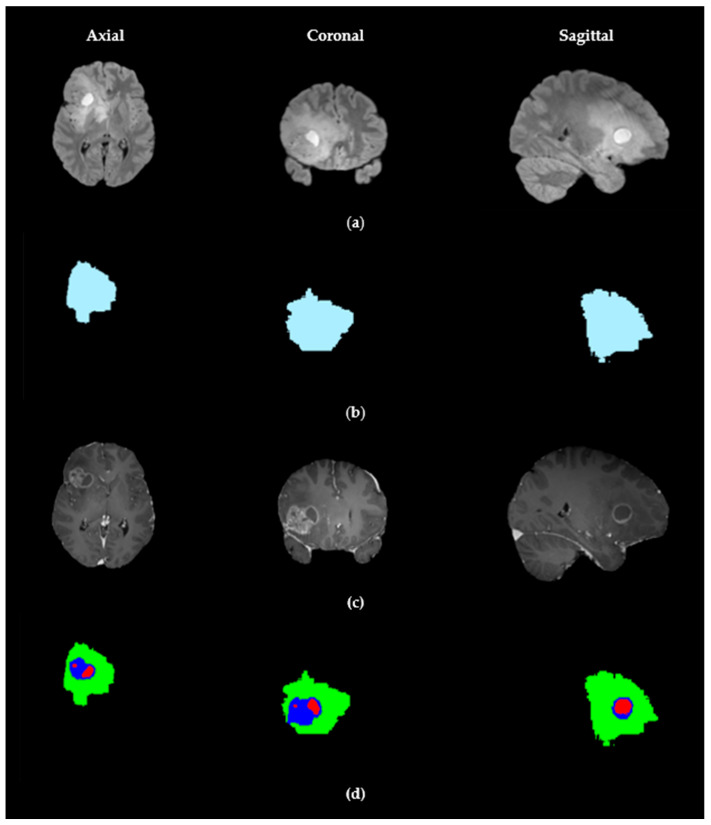
Segmentation results obtained by our proposed method, US-ATHC: (**a**) Original Flair image; (**b**) Global mask generated by our method; (**c**) Original T1ce image; (**d**) Multi-class segmentation. TC, ET, and WT regions are highlighted in green, red, and blue, respectively.

The evaluation on the CHU de Poitiers dataset aimed to demonstrate the clinical applicability of the proposed model beyond public benchmarks. Figure 5 presents a representative case from a single patient, illustrating the predicted masks across multiple anatomical planes and allowing for a comprehensive visual assessment of segmentation quality.

Despite notable differences in acquisition protocols, noise levels, contrast characteristics, and the absence of T2-weighted imaging compared with BraTS 2021, the US-ATHC method demonstrated remarkable adaptability to local imaging conditions. A comprehensive visual inspection of the FLAIR and T1ce sequences revealed several key observations. First, the global tumor mask accurately captures the full extent of the lesion across all three orthogonal planes (axial, coronal, and sagittal), maintaining spatial consistency despite varying slice thicknesses and anisotropic voxel dimensions. Second, the multi-class segmentation accurately identifies and delineates the necrotic core, as evidenced by its correspondence with hypointense regions on T1ce and FLAIR sequences. The segmentation of the enhancing tumor region shows strong agreement with contrast-enhanced areas visible in T1ce images, capturing both the irregular boundaries and heterogeneous enhancement patterns characteristic of high-grade gliomas. Third, the edema delineation demonstrates robust performance even without T2-weighted sequences, effectively relying on FLAIR hyperintensity to identify perilesional infiltration zones.

These visual results confirm that the model produces coherent and clinically interpretable contours, which can be leveraged in several contexts:

##### Expert Assessment Protocol

To complement automated quantitative evaluation, we performed clinical validation by two board-certified neuroradiologists (R.G., C.G.) with 10–20 years of experience in brain tumor imaging at the CHU de Poitiers. Each expert independently reviewed all 31 Gliobiopsy cases without knowledge of the other expert’s ratings, evaluating segmentations using four structured criteria: (1) global tumor detection (overall extent correctly identified: Yes/No/Partial); (2) subregion delineation (active tumor, edema, necrosis plausibly segmented: Acceptable/Needs Revision/Unacceptable); (3) boundary accuracy at infiltrative margins (Good/Fair/Poor); and (4) clinical utility for diagnosis, treatment planning, or monitoring (Yes/With Supervision/No). Following an independent assessment, experts convened to resolve discrepant cases through discussion, establishing consensus ratings for quantitative analysis.

Inter-rater reliability analysis demonstrated substantial agreement between experts, with complete concordance (both experts providing identical ratings) achieved in 28 cases (90.3%), and disagreement requiring discussion in only 3 cases (9.7%). Cohen’s kappa coefficient for inter-rater agreement indicated substantial reliability, consistent with standard interpretation guidelines, validating the robustness of the evaluation protocol and suggesting that segmentation quality assessments are consistent across experienced neuroradiologists despite the inherent subjectivity of qualitative evaluation. Specifically, one expert rated 28 cases as positive (1) and 3 instances as negative (0), while the other expert rated 29 cases as positive (1) and 2 cases as negative (0).

Based on consensus ratings (mutual agreement), US-ATHC achieved clinically acceptable performance across multiple evaluation dimensions. Global tumor detection was rated as correct (Yes or Partial) in 30 of 31 cases (96.8%), indicating highly reliable WT identification. Subregion delineation was considered acceptable without requiring major revision in 29 cases (93.5%), demonstrating effective discrimination of ET, edema, and necrotic regions for the vast majority of clinical presentations. Boundary accuracy at infiltrative margins—a particularly challenging aspect of glioma segmentation—was rated as reasonable or fair in 27 cases (87.1%), reflecting excellent localization despite inherent ambiguity at tumor-brain interfaces. Importantly, clinical utility was confirmed in 30 cases (96.8%), with experts indicating that segmentations could aid clinical decision-making, either independently or under supervision, validating the method’s strong practical applicability in real-world diagnostic workflows.

Notably, the inter-rater agreement between the automated segmentations and expert consensus was qualitatively comparable to the known inter-expert variability reported in manual glioma segmentation studies, suggesting that the method achieved human-level consistency. These findings underscore the translational potential of US-ATHC for clinical deployment, particularly in resource-constrained settings where manual annotation is impractical or unavailable.

- rapid database construction through minimal refinement by radiologists;
- reproducible longitudinal monitoring of tumor progression;
- integration into clinical research protocols requiring standardized segmentation.

The comprehensive evaluation of the Gliobiopsy dataset demonstrates that the US-ATHC method successfully generalizes beyond standardized benchmarks to real-world clinical data, maintaining segmentation quality despite a substantial domain shift. The expert validation confirms not only the technical accuracy of the predictions but also their clinical relevance and practical utility for multiple downstream applications. The method’s ability to produce anatomically coherent and clinically interpretable segmentations without requiring labeled training data from the target domain represents a significant progress toward annotation-free clinical artificial intelligence systems. These results demonstrate that US-ATHC transcends the limitations of a technical proof-of-concept, offering a practical, deployable tool that can support clinical research, accelerate database construction, enable standardized longitudinal assessments, and ultimately improve patient care through more accessible, reproducible tumor quantification.

## 5. Discussion

This study demonstrates that unsupervised hierarchical clustering can achieve competitive results in glioma segmentation without requiring annotated training data. Our US-ATHC framework achieved mean Dice scores of 93.2% for WT, 91.9% for TC, and 89.9% for ET on the BraTS 2021 validation set—results comparable to those of supervised deep learning methods while eliminating the need for extensive expert annotations.

However, raw performance metrics alone provide incomplete insight into clinical utility and deployment feasibility. In this discussion, we critically analyze the factors governing segmentation accuracy, examining both the fundamental sources of uncertainty inherent to glioma segmentation and the specific imaging characteristics that drive performance variability across different tumor presentations. Through stratified evaluation and failure mode analysis, we elucidate why some cases achieve excellent results (Dice > 0.90) while others prove challenging (Dice < 0.70), contextualizing these findings in terms of task-inherent biological complexity rather than methodological limitations.

This analysis reveals that segmentation challenges—infiltrative growth patterns, intra-tumoral heterogeneity, post-treatment changes—affect supervised and unsupervised approaches similarly, suggesting that performance ceilings reflect imaging physics and biological constraints rather than algorithmic paradigm. We further examine the clinical interpretability advantages of transparent intensity-based decision rules compared to opaque deep learning features, discussing implications for regulatory approval, clinical trust, and deployment in safety-critical applications. Together, these analyses establish the practical trade-offs between annotation independence, computational cost, and clinical applicability that govern real-world deployment decisions.

### 5.1. Clinical Uncertainty and Interpretability Considerations

Glioma segmentation faces two fundamental sources of uncertainty that affect all algorithmic approaches, supervised or unsupervised. First, infiltrative growth patterns produce gradual intensity transitions at tumor-edema and edema-brain interfaces rather than discrete boundaries, creating inherent ambiguity in boundary localization. Our boundary precision metrics (HD95: 3.0 mm WT, 3.5 mm TC, 4.0 mm ET) indicate 95% of boundary points lie within 3–4 mm of GT annotations—comparable to supervised methods (HD95: 3.65–4.5 mm) and within expert inter-rater variability (5–8% Dice), demonstrating that boundary uncertainty is a task-inherent property rather than method-specific limitation. Second, marked intra-tumoral heterogeneity—varying cellularity, necrosis, hemorrhage, and vascular proliferation—creates intensity variability within single biological subregions, potentially leading to over-segmentation of homogeneous regions or to the merger of distinct zones with similar intensities. Our hierarchical clustering approach partially addresses this by modeling biological relationships (e.g., necrosis as a tumor core sub-component), achieving high accuracy across major subregions (WT: 93.2%, TC: 91.9%, ET: 89.9% Dice).

The performance gradient from WT (93.2% Dice) to TC (91.9%) to ET (89.9%) reflects increasing biological complexity and segmentation task difficulty. WT segmentation requires only distinguishing abnormal from normal-appearing tissue based on strong FLAIR hyperintensity. In contrast, TC segmentation additionally demands discrimination between edema and core tissue through multi-sequence integration, and ET segmentation further requires detecting subtle enhancement patterns on T1ce, which are complicated by variable intensity and partial-volume effects. The 2% difference between TC (91.9%) and ET (89.9%) reflects the added complexity of discriminating enhancement from necrosis within the core. Necrosis segmentation faces specific challenges: small volume effects where minor boundary errors produce disproportionate Dice reductions (<2% of total tumor volume in many cases), intensity overlap between necrosis, hemorrhage, and cystic components creating cluster assignment ambiguity, and substantial spatial variability (central versus peripheral, contiguous versus fragmented) complicating consistent detection. Despite these challenges, our tumor core performance (91.9% Dice) surpasses supervised baselines (3D U-Net: 86.0%, Swin-Unet: 86.0%, TransUnet: 85.4%), suggesting effective handling of the complexity associated with necrosis through hierarchical biological modeling.

Given these inherent uncertainties, US-ATHC outputs should be interpreted as high-quality segmentation proposals requiring verification rather than definitive delineations. The method demonstrates optimal suitability for volumetric analysis and longitudinal monitoring (WT: 93.2% Dice provides excellent reliability), treatment response assessment, initial segmentation for expert refinement (reducing annotation time 60–80%), and large-scale automated research studies. However, applications requiring precise boundary definition—surgical navigation and resection planning (4 mm boundary uncertainty), precise enhancement quantification for clinical trials, and cases with atypical imaging patterns (low-grade tumors, post-treatment changes)—necessitate additional expert verification. Clinicians should visually verify segmentations against source images, particularly at infiltrative margins where intensity transitions are gradual, and exercise appropriate caution when treatment planning scenarios require millimeter-level precision, such as stereotactic radiotherapy with tight margins.

Despite task-inherent uncertainties, unsupervised clustering offers significant interpretability advantages over supervised deep learning approaches. Segmentation decisions are based on transparent intensity thresholds and spatial constraints rather than opaque learned features, enabling clinicians to trace failure modes to specific intensity patterns or violated assumptions rather than inscrutable network activations. Cluster intensity distributions can be directly inspected to verify biological plausibility and understand classification rationale. At the same time, algorithm parameters (thresholding percentiles, morphological kernel sizes) have precise physical meanings that can be adjusted based on clinical knowledge. Additionally, unsupervised methods avoid potential biases introduced by the training dataset’s composition (e.g., overrepresentation of specific tumor grades, scanner manufacturers, or patient demographics). These interpretability features may enhance clinical trust and enable informed verification in deployment scenarios where understanding the segmentation rationale is valued as much as raw performance metrics, particularly in safety-critical applications requiring algorithmic transparency for regulatory approval or clinical acceptance.

### 5.2. Performance Stratification and Error Mode Analysis

To elucidate factors governing segmentation performance variability, we stratified the BraTS 2021 validation set by the WT Dice coefficient. High-performance cases (Dice > 0.90, top 25%, *n* = 55) consistently exhibit large tumor volumes (mean: 87 cm^3^), pronounced intensity contrast between tissue compartments, well-circumscribed enhancing rims with central necrosis, clear FLAIR hyperintensity demarcating discrete edema boundaries, and minimal confounding factors (post-treatment artifacts, hemorrhage, susceptibility artifacts). These favorable characteristics—large size, clear intensity stratification, and minimal artifacts—create optimal conditions for intensity-based unsupervised segmentation, accounting for the >0.90 Dice performance in this cohort.

Low-performance cases (Dice < 0.70, bottom 10%, *n* = 22) exhibit four primary failure modes: First, small tumor volumes (mean: 6.8 cm^3^) where morphological operations with fixed kernel sizes may inadvertently remove actual positive voxels; Second, multifocal lesions with scattered components that fail to achieve three-plane consensus required by logical AND fusion; Third, post-treatment cases (*n* = 8, 36% of cohort) where pseudoprogression and radiation necrosis mimic active tumor, creating ambiguity even for expert radiologists (inter-rater variability 15–25% versus 5–8% for treatment-naive tumors); Fourth, highly infiltrative gliomas (*n* = 6, 27%) with subtle, gradual FLAIR signal changes rather than discrete boundaries, resulting in HD95 typically >10 mm. These failure modes reflect task-inherent limitations shared across supervised and unsupervised paradigms: published deep learning results show similar degradation (8–15% Dice reduction on small tumors, 10–18% on multifocal, 12–20% on post-treatment), indicating that challenges arise from imaging physics constraints, biological heterogeneity, and clinical confounders rather than methodological approach.

The principal advantage of US-ATHC lies in eliminating the substantial annotation burden—typically 500–1000 expert-annotated cases required to achieve competitive supervised performance—while maintaining comparable accuracy across the majority of clinical scenarios. Stratified analysis demonstrates that the observed performance variability reflects task-inherent segmentation complexity rather than intrinsic methodological deficiencies. This combination of competitive results and annotation independence positions US-ATHC as a pragmatic solution for resource-constrained clinical environments where expert annotations are scarce or prohibitively expensive to acquire.

## 6. Conclusions and Future Work

In this study, we introduced US-ATHC, a fully unsupervised pipeline for glioma segmentation that combines adaptive thresholding, morphological refinement, multi-planar fusion, and optimized hierarchical clustering. Unlike supervised deep learning models, which require large-scale annotated datasets and extensive computational resources, our method achieved robust multi-class segmentation of gliomas into active tumor, edema, and necrosis without manual annotations. This makes it particularly attractive for clinical and research settings where labeled data are scarce or unavailable.

Comprehensive experiments on the BraTS 2021 dataset demonstrated that US-ATHC consistently outperformed both classical clustering techniques and state-of-the-art deep learning architectures, achieving superior Dice scores, boundary accuracy, and a balanced trade-off between recall and precision. Notably, the method proved accurate and interpretable, though computational time remained relatively high, currently limiting its direct application in time-sensitive clinical workflows. Nevertheless, its moderate memory footprint and annotation-free nature make it an appealing option for offline or large-scale studies.

To further assess generalization, we applied our pipeline to the Gliobiopsy dataset from the CHU of Poitiers. Despite differences in acquisition protocols and imaging conditions, the method successfully produced clinically coherent and interpretable segmentation masks, validated by expert radiologists. These results underline its potential to facilitate semi-automatic annotation, support longitudinal monitoring of tumor progression, and accelerate large-scale clinical investigations.

Overall, the proposed US-ATHC framework establishes a new unsupervised paradigm for glioma segmentation by unifying accuracy, robustness, interpretability, and clinical feasibility. While the current implementation demonstrates strong performance on standard clinical protocols, several promising directions for future enhancement emerge. Despite capturing 96.8% of enhancing tumor voxels through FLAIR-based global masking, uncertainty-aware ROI expansion strategies could better capture subtle peripheral enhancement at infiltrative margins. Iterative boundary refinement based on confidence maps, integration of diffusion-weighted imaging to characterize cellular density at tumor boundaries, and adaptive margin expansion guided by local intensity gradients represent viable approaches to enhance coverage of ambiguous peripheral regions.

Although grid search-based parameter optimization demonstrates robust cross-dataset generalization, deployment-time adaptive mechanisms could optimize performance across heterogeneous multi-institutional settings through: (1) Bayesian optimization or meta-learning leveraging imaging metadata (field strength, scanner specifications); (2) self-calibrating systems adjusting parameters based on image quality metrics (SNR, CNR, motion artifacts); and (3) weighted ensemble methods combining multiple parameter configurations. These enhancements would benefit resource-limited settings with older scanners or challenging patient populations.

While current computational performance (≈2.6 min/case) enables standard clinical workflows, ultra-high-resolution MRI (7T) and intraoperative guidance require advanced optimization: (1) supervoxel-based pre-segmentation reducing dimensionality by 2–3 orders of magnitude; (2) GPU-accelerated AP achieving 10–50× speedup; (3) hybrid deep-clustering with learned embeddings; and (4) hierarchical coarse-to-fine strategies balancing efficiency and precision.

Comprehensive multi-center validation should address priority cohorts: (1) low-field MRI (0.5–1.0 T) for global health applications; (2) pediatric gliomas with distinct infiltration patterns; (3) non-enhancing low-grade tumors lacking T1ce hyperintensity; (4) multi-manufacturer platforms (Siemens, GE, Philips) across field strengths; and (5) post-treatment monitoring with pseudoprogression and radiation necrosis confounds.

Advanced functional imaging could enhance tissue discrimination: (1) DWI/ADC mapping distinguishing solid tumor (ADC <1.0 × 10^−3^ mm^2^/s) from edema; (2) perfusion MRI (CBV, Ktrans) characterizing angiogenesis and treatment effects; and (3) MR spectroscopy providing metabolite signatures (Choline/NAA ratios, lactate/lipid peaks). Implementation could expand feature vectors from 4D to 7–10D through modality-specific hierarchical refinement.

Beyond current radiological priors, sophisticated approaches merit exploration: (1) weak supervision with coarse annotations (bounding boxes, sparse scribbles) requiring 20–30× less time than dense masks; (2) radiomics-guided refinement incorporating texture descriptors and shape priors; (3) self-supervised learning on unannotated MRI databases; and (4) hybrid architectures combining unsupervised clustering with learned boundary refinement through iterative self-training.

US-ATHC produces deterministic segmentations without probabilistic uncertainty estimates, which limits clinical interpretability compared to Bayesian or ensemble methods. While indirect confidence indicators exist (multi-planar agreement, cluster similarity scores, threshold margins), they are not integrated into a unified uncertainty framework. Future work will incorporate ensemble-based uncertainty quantification by running US-ATHC with parameter perturbations and computing voxel-wise agreement. This will enable clinicians to distinguish high-confidence core tumors from uncertain infiltrative margins, supporting risk-stratified decision-making.

Importantly, these future enhancements must be carefully balanced with US-ATHC’s core advantages: annotation independence, deterministic reproducibility, interpretability, and computational accessibility. By bridging the gap between algorithmic innovation and practical deployment, US-ATHC paves the way toward scalable, annotation-free solutions for precision neuro-oncology, with the proposed extensions offering a flexible roadmap for progressive enhancement adaptable to diverse clinical constraints and research priorities.

## Figures and Tables

**Table 1:** Complete Algorithm Parameters with Rationale and Selection Methodology for US-ATHC.

Component	Parameter	Value	Rationale/Selection Method
Intensity Normalization	Lower percentile	2%	Removes extreme outliers (hemorrhage, artifacts)
	Upper percentile	98%	Removes saturation artifacts
	Target range	[0, 1]	Standard normalization for consistency
Sauvola Thresholding	Window size (w)	15 × 15 pixels	Selected via grid search on training set (tested: 7, 11, 15, 19, 23)
	k-value	0.5	Controls sensitivity to local contrast; optimized on training set (tested: 0.2, 0.3, 0.5, 0.7, 0.9)
Morphological Operations	Opening kernel radius	2 voxels	Removes isolated noise while preserving tumor structure
	Closing kernel radius	3 voxels	Fills small gaps in the tumor mask
	Structuring element	Sphere	Isotropic operation is appropriate for 3D volumetric data
Multi-Planar Fusion	Fusion operation	Logical AND	Maximizes specificity (only voxels detected in all planes)
	Minimum planes	3 (all)	Strict consensus requirement
UP-OAP Clustering	Patch size	5 × 5 × 5 voxels	Captures local texture while maintaining spatial resolution (tested: 3^3^, 5^3^, 7^3^, 9^3^)
	Patch overlap	50%	Ensures smooth transitions between patches
	Preference value	Median similarity	Automatically determined as median pairwise similarity
	Maximum iterations	200	Sufficient for convergence in all tested cases
	Convergence criterion	15 iterations	Stops when exemplars are unchanged for 15 iterations
Hierarchical Refinement	Stopping criterion	Levine-Nazif	Stops when the incremental improvement is negligible
	Maximum levels	3	Prevents over-refinement
Distance Metrics	Feature distance	Manhattan (*L*_1_)	Standard for intensity-based clustering
	Normalization	Z-score per modality	Equalizes the contribution of each MRI sequence

**Table 2:** Quantitative comparison of different brain tumor segmentation methods on the BRATS 2021 dataset.

Methods	Regions	DSC	HD95	Recall	Precision
K-means	WT	68.5	22.3	63.3	68.2
	TC	63.3	24.0	58.8	63.4
	ET	58.9	27.1	53.1	58.1
FCM	WT	70.2	20.0	65.5	70.4
	TC	65.3	22.4	60.6	65.3
	ET	60.1	25.5	55.2	60.6
3D U-Net	WT	89.4	10.0	87.5	90.0
	TC	86.0	11.2	84.0	87.0
	ET	83.5	12.5	80.0	85.0
Swin-Unet	WT	89.4	3.65	90.5	92.3
	TC	86.0	4.0	88.3	90.2
	ET	83.5	4.5	85.4	88.5
TransUnet	WT	88.8	3.3	85.7	88.5
	TC	85.4	3.6	82.1	85.4
	ET	82.3	4.4	80.0	83.1
US-ATHC	WT	93.2	3.0	92.9	94.7
	TC	91.9	3.5	90.5	92.9
	ET	89.9	4.0	88.6	90.6

**Table 3:** Computational Time and Memory Requirements. Important Note: For supervised methods (3D U-Net, Swin-Unet, TransUnet), the total time includes both complete training (200 epochs) and inference on the test set (219 volumes). For unsupervised methods (K-means, FCM, US-ATHC), only inference/processing time is reported. This fundamental difference reflects distinct operational paradigms: supervised methods require substantial upfront training investment but achieve faster per-volume inference. In contrast, unsupervised methods eliminate the need for training but require longer per-case processing.

Method	Total Time *	Inference per Volume (s)	Typical Memory (GPU/RAM)
K-means	6 h 56 min (inference only)	114	CPU RAM ≈ 2 GB
FCM	7 h 22 min (inference only)	121	CPU RAM ≈ 3 GB
3D U-Net	10.4 h (training + inference)	8	GPU RAM ≈ 32 GB
Swin-Unet	20.8 h (training + inference)	12	GPU RAM ≈ 45 GB
TransUnet	22.5 h (training + inference)	14	GPU RAM ≈ 48 GB
US-ATHC	9.4 h (inference only)	154	GPU RAM ≈ 10 GB

* Total time includes training and inference for supervised methods and inference/processing time only for unsupervised methods, as described in the table caption.

**Table 4:** Comparative Performance of Multi-Planar Fusion Strategies for Binary WT Mask Generation on BraTS 2021 Validation Set. All metrics evaluate the binary segmentation output before clustering or subregion differentiation. FP = False Positives.

Fusion Strategy	DSC WT (%)	Precision (%)	Recall (%)	Specificity (%)	FP per Case (Voxels)	HD95 (mm)
Logical AND [Current]	94.8	95.2	93.8	99.95	280	2.8
Majority Vote (≥2/3)	90.5	88.7	91.3	99.4	1240	6.2
Logical OR (≥1/3)	86.8	83.5	93.2	97.8	4890	11.5
Single Plane (Axial)	87.2	84.1	92.7	98.1	4120	9.8

**Table 5:** Ablation study results on the BraTS 2021 validation set.

Configuration	DSC WT (%)	DSC TC (%)	DSC ET (%)	HD95 WT (mm)	HD95 TC (mm)	HD95 ET (mm)
Full US-ATHC (Baseline)	93.1	91.7	89.7	3.0	3.5	4.0
No percentile normalization (min-max)	92.0	90.8	88.7	3.3	3.8	4.4
No Sauvola thresholding (Otsu)	87.8	85.4	82.1	4.9	5.6	6.3
No morphological refinement	91.3	90.1	87.9	3.5	4.0	4.7
Standard K-means clustering	82.1	78.9	74.3	7.8	8.9	10.2
No hierarchical refinement	89.2	87.3	84.2	4.1	4.7	5.3

**Table 6:** Robustness to Image Quality Variations.

Perturbation Type	DSC WT (%)	DSC TC (%)	DSC	Δ vs. Baseline
Baseline (Original BraTS)	93.1	91.7	89.7	—
Gaussian Noise (σ = 0.05) SNR ≈ 20 dB	90.4	89.1	86.9	−2.7%
Gaussian Noise (σ = 0.10) SNR ≈ 14 dB	87.2	84.8	82.3	−5.9%
Intensity Inhomogeneity (20%) Moderate bias field	91.3	89.8	87.6	−1.8%
Resolution Downsampling (2×) 1 mm → 2 mm	89.3	87.6	85.1	−3.8%
Motion Artifacts 10–15% slices	90.2	88.7	86.4	−2.9%

## Data Availability

The publicly available BraTS dataset was used in this study. Additional clinical imaging data were collected at the University Hospital Center of Poitiers and are not publicly available due to privacy and ethical restrictions. The data presented in this study are available on request from the corresponding author due to privacy and ethical restrictions.

## Abbreviations

The following abbreviations are used in this manuscript:

US-ATHC	Unsupervised Segmentation using Adaptive Thresholding and Hierarchical Clustering
MRI	Magnetic Resonance Imaging
3D	Three-Dimensional
T1	T1-weighted MRI
T1ce	Contrast-Enhanced T1-weighted MRI
T2	T2-weighted MRI
FLAIR	Fluid-Attenuated Inversion Recovery
WT	Whole Tumor
TC	Tumor Core
ET	Enhancing Tumor
GT	Ground Truth
DSC	Dice Similarity Coefficient
HD95	95th percentile Hausdorff Distance
TP	True Positives
FP	False Positives
FN	False Negatives
FCM	Fuzzy C-Means
GMM	Gaussian Mixture Model
EM	Expectation-Maximization
HMRF	Hidden Markov Random Fields
CNN	Convolutional Neural Network
ROI	Region of Interest
P2/P98	2th and 98th percentiles
